# Effects of Alkyl Spacer Length in Carbazole‐Based Self‐Assembled Monolayer Materials on Molecular Conformation and Organic Solar Cell Performance

**DOI:** 10.1002/advs.202410277

**Published:** 2024-12-04

**Authors:** Qiaonan Chen, Kangbo Sun, Leandro R. Franco, Jingnan Wu, Lars Öhrström, Xianjie Liu, Maureen Gumbo, Mailde S. Ozório, C. Moyses Araujo, Guangye Zhang, André Johansson, Ellen Moons, Mats Fahlman, Donghong Yu, Yufei Wang, Ergang Wang

**Affiliations:** ^1^ Department of Chemistry and Chemical Engineering Chalmers University of Technology Göteborg SE‐412 96 Sweden; ^2^ College of New Materials and New Energies Shenzhen Technology University Shenzhen 518118 China; ^3^ Department of Engineering and Physics Karlstad University Karlstad 65188 Sweden; ^4^ Department of Science and Technology Laboratory of Organic Electronics (LOE) Linköping University Norrköping 60174 Sweden; ^5^ Department of Chemistry University of Copenhagen Universitetsparken 5 Copenhagen DK‐2100 Denmark; ^6^ Materials Theory Division Department of Physics and Astronomy Uppsala University Uppsala 75120 Sweden; ^7^ Departmetnt of Chemistry and Bioscience Aalborg University Aalborg DK‐9220 Denmark; ^8^ Sino‐Danish Center for Education and Research Aarhus DK‐8000 Denmark; ^9^ School of Materials Science and Engineering Zhengzhou University Zhengzhou 450001 China

**Keywords:** alkyl spacer length, intermolecular interaction, molecular conformation, self‐assembled monolayer (SAM), single crystals

## Abstract

Carbazole‐based self‐assembled monolayer (SAM) materials as hole transport layers (HTL) have led organic solar cells (OSCs) to state‐of‐the‐art photovoltaic performance. Nonetheless, the impact of the alkyl spacer length of SAMs remains inadequately understood. To improve the knowledge, four dichloride‐substituted carbazole‐based SAMs (from 2Cl‐2PACz to 2Cl‐5PACz) with spacer lengths of 2–5 carbon atoms is developed. Single crystal analyses reveal that SAMs with shorter spacers exhibit stronger intermolecular interactions and denser packing. The molecular conformation of SAMs significantly impacts their molecular footprint and coverage on ITO. These factors result in the highest coverage of 2Cl‐2PACz and the lowest coverage for 2Cl‐3PACz on ITO. OSCs based on PM6:L8‐BO with 2Cl‐2PACz as HTL achieved high efficiencies of 18.95% and 18.62% with and without methanol rinsing of the ITO/SAMs anodes, corresponding to monolayer and multilayer structures, respectively. In contrast, OSCs utilizing the other SAMs showed decreased efficiencies as spacer length increased. The superior performance of 2Cl‐2PACz can be attributed to its shorter spacer, which reduces series resistance, hole tunneling distance, and barrier. This work provides valuable insights into the design of SAMs for high‐performance OSCs.

## Graphical abstract

The spacer length in carbazole‐based self‐assembled monolayer (SAM) hole‐transport layer materials significantly influences the hole tunneling distance, which in turn affects the efficiency of organic solar cells. Additionally, spacer length impacts the molecular conformation, as revealed by single crystal analysis, resulting in varied coverage on indium tin oxide (ITO) surfaces.

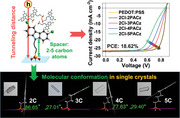

## 1 Introduction

Organic solar cells (OSCs) have attracted extensive research interests due to their lightweight, mechanical flexibility, semitransparency, and solution processability for large‐area manufacturing.^[^ ^1^ ^]^ To realize OSCs with both high power conversion efficiency (PCE) and stability, significant efforts have been devoted to the development of innovative polymers,^[^ ^2^ ^]^ small molecules^[^ ^3^ ^]^ and additives^[^ ^4^ ^]^ for the active layer. These endeavors have successfully elevated the PCEs of polymer donor/small molecule acceptor, all polymer systems, and ternary systems up to 19.70%,^[^ ^5^ ^]^ 19.06%,^[^ ^6^ ^]^ and 20.17%,^[^ ^7^ ^]^ respectively. On the other hand, interfacial layers also play key roles in enhancing PCE by reducing energy barrier, facilitating charge carrier extraction from the active layer to the corresponding electrodes, and improving device stability through applying neutral and hydrophobic interfacial materials.^[^ ^8^ ^]^ In contrast to intensively studied active layer materials, there are multiple opportunities in directing attention toward new interfacial layer materials.

In the past, most efforts have been given to explore the electron‐transport layer (ETL) materials, such as ZnO,^[^ ^9^ ^]^ PFN‐Br,^[^ ^10^ ^]^ PNDIT‐F3N,^[^ ^11^ ^]^ PDINO,^[^ ^12^ ^]^ PDINB,^[^ ^13^ ^]^ and PDINN,^[^ ^14^ ^]^ which have been successfully applied in different solar cell systems (full names are listed in Supporting Information). On the contrary, the selections of hole‐transport layer (HTL) materials are still relatively limited. PEDOT: PSS and MoO_3_ are the most widely used ones due to their steady initial device performances. However, the acidic nature of PEDOT: PSS, which degrades the indium‐tin‐oxide (ITO) electrode, combined with its sensitivity to moisture, results in poor long‐term stability of devices. In the case of MoO_3_ layers, energy‐consuming vacuum techniques are required for their processing, limiting their compatibility with large‐scale manufacturing.^[^ ^15^ ^]^ Thus, the development of innovative HTL materials featuring simple structures, suitable energy levels, and superior stability is urgently demanded. Self‐assembled monolayer (SAM) materials rose among HTL materials distinguished by their unique advantages: 1) their simple structures which allow easy modifications for a fine‐tuning of energy levels to align with active layer, thus facilitating ohmic contact and improving hole carrier transport;^[^ ^16^ ^]^ 2) their high lowest unoccupied molecular orbital (LUMO) can effectively block electrons transferring to anodes, reducing charge recombination; 3) their amendable surface smoothness and wettability makes it compatible to different types of photovoltaics including perovskite solar cells (PSCs) and OSCs.^[^ ^17^ ^]^

**Figure 1:**
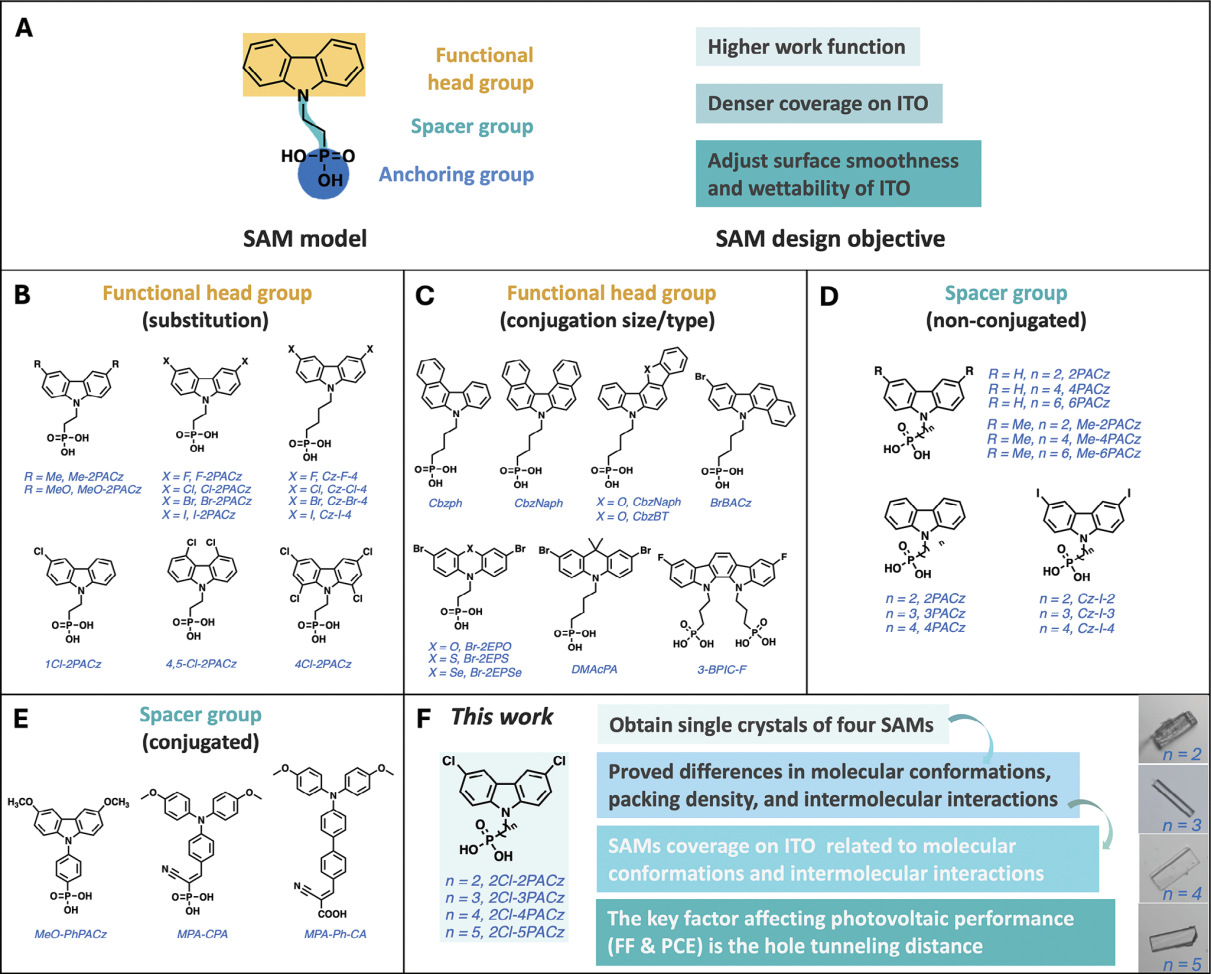
(A) SAM model and design objectives. Structures of previously reported SAMs with different (B,C) functional head groups and (D,E) spacer groups. (F) The SAMs structures, microscope images of single crystals, and novelty in this work.

A typical SAM contains three components, including a functional headgroup, a spacer group, and an anchoring group (**Figure** **1**A). Recently, SAMs comprising carbazole,^[^ ^7^, ^16^, ^18^ ^]^ phenothiazine,^[^ ^17^, ^19^ ^]^ and their derivatives^[^ ^16^, ^18^, ^19^, ^20^ ^]^ as functional headgroups exhibit excellent photovoltaic (PV) properties in both PSCs and OSCs. Great efforts have been devoted to modifying substituents^[^ ^16^, ^18^, ^21^ ^]^ and conjugation size^[^ ^5^, ^16^, ^20^, ^22^ ^]^ of carbazole moieties, which effectively optimize work functions (WF) of bottom ITO substrate and their interaction with upper active layers, achieving impressive PCEs of over 20%^[^ ^7^ ^]^ for OSCs and near 26%^[^ ^20a^ ^]^ for PSCs (Figure 1B,C). Nowadays, phosphonic acid (PA)‐based anchoring groups are the most widely used in OSCs and PSCs due to their multiple binding sites and stronger hydrogen ions dissociation capability (pK_a1_ = 1–6, pK_a2_ = 5–11) over other anchors (e.g., ─OH, ─SH, ─COOH, ─Si (OR)_3_, and ‐SiCl_3_). This allows PA anchor to form very stable covalent bonds with ITO surface.^[^ ^23^ ^]^ However, it is noteworthy that, compared to the detailed research on the influence of structural modification of carbazole on the PV performance, studies on the effect of alkyl spacer length of carbazole‐derivative‐based SAMs are limited (Figure 1D).^[^ ^18^, ^21^, ^24^ ^]^ Particularly, the impact of alkyl spacer length on molecular conformation, intermolecular interaction, coverage density of SAMs on ITO surfaces, WF of SAMs‐modified ITO, and their PV performance in OSC systems, remains not well‐understood. Therefore, addressing these doubts and conducting further structural analyses on single crystals of SAMs are crucial for understanding the underlying structure‐performance relationship. Moreover, the layer count in spin‐coated ITO/SAM films—whether untreated or rinsed with solvents or SAM solutions—and its impact on PV performance must be further elucidated. Specifically, understanding how SAM layer thickness influences hole tunneling and extraction is essential for optimizing device efficiency.

Herein, we developed a series of dichloride‐substituted carbazole‐based SAM materials with different alkyl spacer lengths (2–5 carbon atoms), denoted as 2Cl‐2PACz,^[^ ^16c^ ^]^ 2Cl‐3PACz, 2Cl‐4PACz, and 2Cl‐5PACz, respectively (Figure 1F). UV–vis spectroscopy revealed that ITO/SAM layers, whether untreated or rinsed with SAM solutions, tend to form multilayer structures. In contrast, methanol‐rinsed ITO/SAM layers yield true monolayers. Single crystal analyses demonstrated that SAMs with shorter spacers exhibit stronger intermolecular interactions and denser packing, while SAMs with longer spacers introduce additional H···H interactions between spacer chains. Carbazole orientations relative to the three oxygen atoms plane in the PA group were measured as 86.65°, 27.01°, 77.63°, and 29.40° for 2Cl‐2PACz, 2Cl‐3PACz, 2Cl‐4PACz, and 2Cl‐5PACz, respectively, which likely affecting molecular footprint and coverage on ITO. The more perpendicular carbazole orientation in 2Cl‐2PACz minimized surface occupation, achieving the highest ITO coverage, while 2Cl‐3PACz showed the lowest coverage due to its larger footprint. H···H interactions between spacers promoted intermolecular arrangement, leading to moderate surface coverage for 2Cl‐4PACz and 2Cl‐5PACz. To study the influence of alkyl spacer length in SAMs on PV performance, we incorporated ITO/SAMs into a range of state‐of‐the‐art OSCs (PM6:L8‐BO, PM6:BTP‐eC9, PM6:Y6). We demonstrated that an increase of spacer length resulted in a decrease of both FF and PCE. Device analyses revealed that the shorter spacer in 2Cl‐2PACz reduced both series and device resistance, enhanced charge transfer, and extended carrier lifetime. This improvement was primarily due to its shorter tunneling distance and lower tunneling barrier in both multilayer and monolayer structures compared to other SAMs, resulting in high PCEs of 18.62% (untreated) and 18.95% (methanol‐rinsed), respectively. This study provides new insights by leveraging single‐crystal perspectives to elucidate how alkyl spacer length in SAMs influences molecular conformation, surface coverage, and PV efficiency in OSCs.

## 2 Results and Discussion

### 2.1 Structures and Thermal Stability of SAMs and the Effect of Concentration on Layer Number on ITO Surfaces

**Figure 2:**
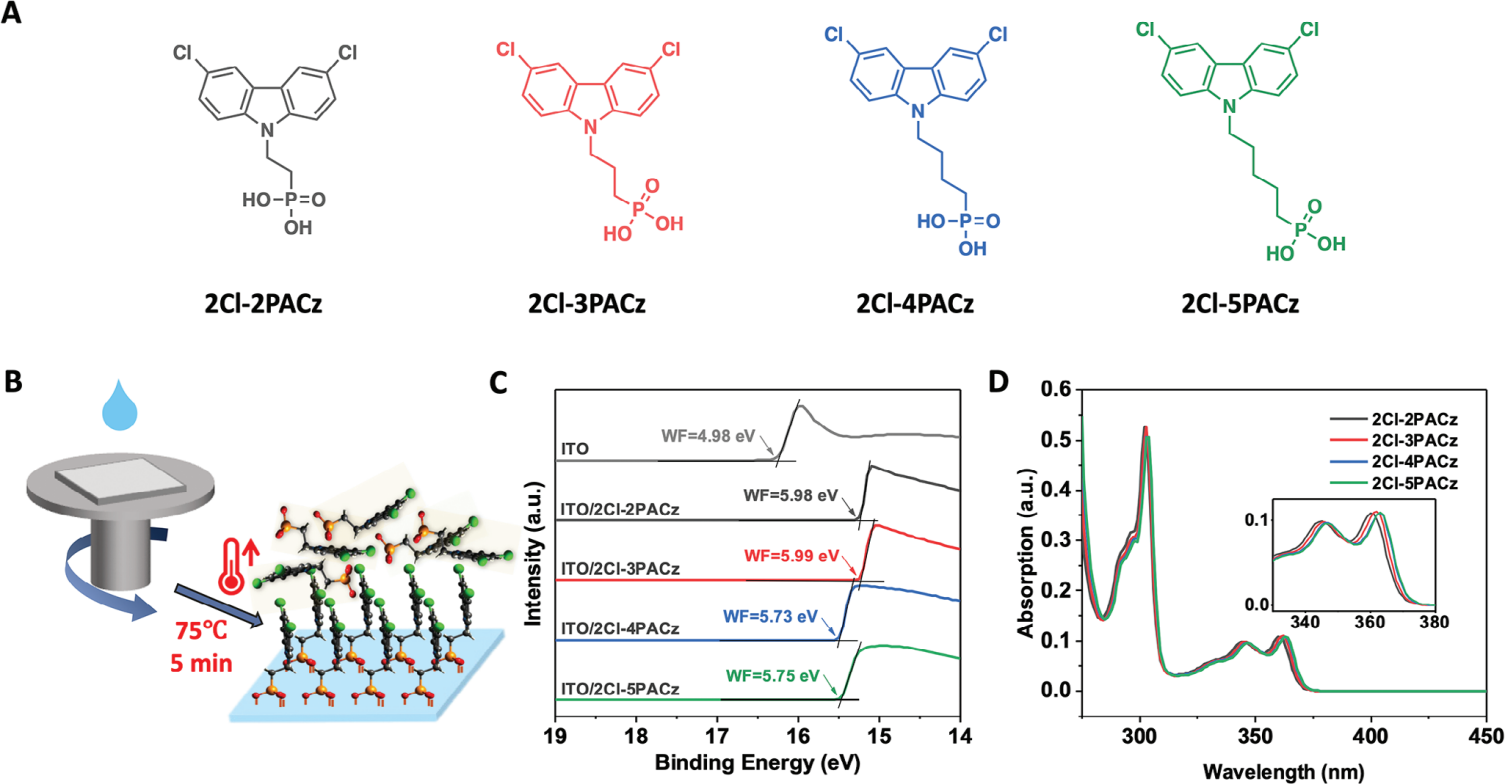
(A) Structures of various SAMs, 2Cl‐2PACz, 2Cl‐3PACz, 2Cl‐4PACz, and 2Cl‐5PACz. (B) The scheme for the preparation of SAMs‐modified ITO glass. (C) UPS spectra (He I lamp with the photo energy of 21.22 eV) of bare‐ and SAMs‐modified ITO for direct determination of the WF. (D) UV–vis absorption spectra of SAM molecules in their ethanol solutions (3 × 10^−5^ M).

The chemical structures of SAM materials investigated in this study (2Cl‐2PACz, 2Cl‐3PACz, 2Cl‐4PACz, and 2Cl‐5PACz) are illustrated in **Figure** **2**A and the details of synthetic procedures are provided in Supporting Information. 2Cl‐2PACz was previously reported by Anthopoulos et al. with its name of Cl‐2PACz, with higher PV performance than those with other halogenations of F, Br, and I.^[^ ^16c^ ^]^ Thanks to their excellent optimization, we selected di‐chlorinated carbazole headgroup in this work. The thermal stability of SAMs molecules was evaluated by thermogravimetric analysis (TGA), with degradation temperatures (5% weight loss) for 2Cl‐2PACz, 2Cl‐3PACz, 2Cl‐4PACz, and 2Cl‐5PACz of 337, 339, 338, and 366 °C, respectively (Figure S1 and Table S1, Supporting Information). These results highlighted their excellent thermal stability suitable for a wide range of optoelectronic applications. As shown in Figure 2B, the preparation of SAMs on ITO surfaces involved facile spin‐coating using a 0.3 mg mL⁻¹ SAMs solution in methanol, followed by annealing at 75 °C for 5 min without additional processing.^[^ ^7^, ^25^ ^]^ It has been demonstrated that the reaction between the PA groups and the hydroxyl groups on the ITO surface results in robust bidentate^[^ ^26^ ^]^ or tridentate^[^ ^16^, ^27^ ^]^ binding configurations on the ITO substrates. Additionally, through comparing the UV–vis spectra of 2Cl‐2PACz spin‐coated onto quartz glass at different concentration, we can infer that the quartz/2Cl‐2PACz films contained ≈1–3 layers, 2–4 layers, and 3–5 layers for samples prepared from concentrations of 0.1, 0.2, and 0.3 mg mL⁻¹, respectively (Figure S2, Supporting Information). However, when the SAMs concentration exceeded 0.4 mg mL⁻¹, the film is relatively thick, which caused strong reflections. Notably, only the first layer is covalently bonded to the quartz surface, making it resistant to methanol washing. In contrast, the additional layers, which are attached to the first layer through Van der Waals interactions, can be easily removed with methanol. This was demonstrated in UV–vis spectra using the precursor molecule 4a of 2Cl‐2PACz, which lacks the PA group and can be entirely washed away by methanol (Figure S3, Supporting Information). Therefore, we infer that only the first layer of SAMs forms a more ordered arrangement, with the PA groups establishing stable bidentate or tridentate binding configurations on the quartz/ITO substrate. On the other hand, the layers above likely adopt a more random packing structure (Figure 2B).^[^ ^17b^ ^]^

### 2.2 Work Function and DFT Simulation

Furthermore, the WF of bare ITO and ITO/SAMs electrodes were measured by ultraviolet photoelectron spectroscopy (UPS) (Figure 2C). The WF of pre‐cleaned bare ITO glass substrates used in our work was 4.98 eV, which is higher than the commonly reported value of ≈4.70 eV.^[^ ^24^, ^28^ ^]^ This increase is likely attributed to the incorporation of fluorine into the ITO glass, as supported by high‐resolution X‐ray photoelectron spectra (HR‐XPS) analysis (Figure S11A, Supporting Information). As expected, SAMs‐modified ITO glass showed significantly enhanced WF.^[^ ^16c^ ^]^ Specifically, 2Cl‐2PACz‐ and 2Cl‐3PACz‐modified ITO surfaces yielded WFs of 5.98 and 5.99 eV, respectively, surpassing those of 5.73 and 5.75 eV for 2Cl‐4PACz‐ and 2Cl‐5PACz‐modified ones. A higher WF is beneficial for hole extraction in ITO‐based OSC devices.^[^ ^16^, ^22^ ^]^ To explore the underlying reasons for the WF distinction, density functional theory (DFT) calculations of the WF for SAM molecules adsorbed on a (111)‐terminated ITO surface were conducted (Figures S4 and S5, Supporting Information).^[^ ^16c^ ^]^ However, the DFT calculations of the WF for SAMs with the most stable conformation in a monolayer on the ITO surface exhibited a different trend from experimental results. The simulation results indicated that 2Cl‐2PACz and 2Cl‐4PACz with even‐numbered carbon atom spacers showed higher WFs (5.41 and 5.45 eV) than 2Cl‐3PACz and 2Cl‐5PACz with odd‐numbered linkers (5.09 and 5.11 eV). This was found related with their simulated molecular conformations on the ITO surface. 2Cl‐2PACz and 2Cl‐4PACz exhibited a more perpendicular orientation of carbazole relative to the ITO surface, which produced a larger vertical component of the molecular dipole moment and therefore resulted in higher WF (Figures S5 and S6, Supporting Information). Notably, the calculated WF was based on the most stable conformations of SAMs in the ordered monolayer on ITO, without considering intermolecular interactions in self‐assembled process^[^ ^23^, ^29^ ^]^ or post‐annealing process,^[^ ^30^ ^]^ which may affect carbazole orientation and SAMs packing density. Furthermore, the dependence of WF on carbazole orientation was calculated. The maximum WF occurred when the carbazole plane was perpendicular to the ITO surface, while a minimum WF was reached when the carbazole plane was parallel to the ITO surface (Figure S7, Supporting Information). Moreover, slight variations in conformation and anchoring position affected the WF, while the energy differences between different states do not vary significantly, indicating that WF can be easily altered (Figure S8, Supporting Information).

Herein, we emphasize the critical role of the first monolayer, which exhibits more ordered packing and significantly influences the WF, although the randomly packed layers above it may also contribute. This suggests that in practical situations, 2Cl‐2PACz and 2Cl‐3PACz may exhibit more perpendicular orientation for carbazole relative to ITO surfaces in the first layer compared to 2Cl‐4PACz and 2Cl‐5PACz after self‐assembling and thermal annealing process, resulting in higher WFs.

Furthermore, the absorption spectra of the SAMs molecules in ethanol were measured (Figure 2D). Their structural similarities (from 2Cl‐2PACz to 2Cl‐5PACz) led to closely matched absorption coefficients, varying only from 3.61 × 10^3^ to 3.67 × 10^3^ M^−1^ cm^−1^ (Table S2, Supporting Information). Additionally, subtle red‐shifts for maximum peak absorption wavelength were observed from 2Cl‐2PACz to 2Cl‐5PACz, gradually increasing from 360 to 361, 363, and 363 nm, respectively. These red shifts can be attributed to a slight enhancement in intramolecular electron transfer as the alkyl spacer lengths increase, as corroborated by the trend observed in DFT simulations (Figures S9 and S10, and Table S3, Supporting Information).

### 2.3 Coverage of SAMs on ITO Surfaces

**Figure 3:**
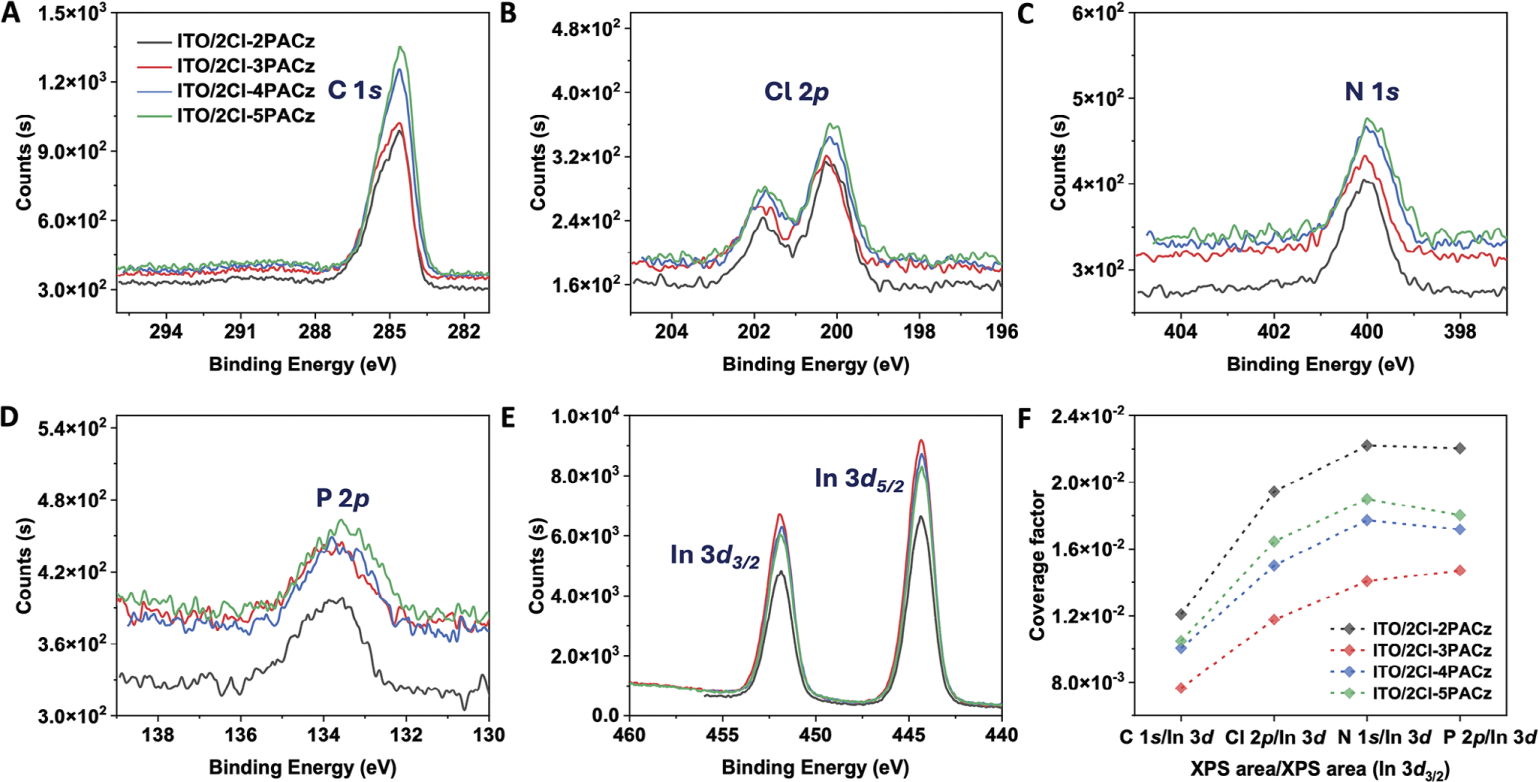
HR‐XPS surveys of the (A) C 1*s*, (B) Cl 2*p*, (C) N 1*s*, (D) P 2*p*, and (E) In 3*d* core levels regions of 2Cl‐2PACz, 2Cl‐3PACz, 2Cl‐4PACz, and 2Cl‐5PACz‐modified ITO. (F) The relative coverage factor of SAMs‐modified ITO, which is calculated by normalizing C 1*s*, Cl 2*p*, N 1*s*, and P 2*p* core level area (divided by the number of atoms) to the In 3*d_3/2_* core level area.

To confirm the formation of SAMs and explore the influence of alkyl spacer length on their coverage on ITO surfaces, HR‐XPS was performed, which can detect an average depth of ≈5 nm. The prominent enhancement in the signal of C 1*s*, alongside the appearance of Cl 2*p*, N 1*s*, and P 2*p* signals in the SAMs‐coated ITO samples, compared to bare ITO, indicated the good formation of SAM layer onto the ITO surface (Figure S11, Supporting Information). Furthermore, peak fitting simulations of C 1*s* spectra showed that the area proportion of the C‐C/C‐H signal roughly increased with alkyl spacer length: from 55 to 54, 68, and 73% for 2Cl‐2PACz, 2Cl‐3PACz, 2Cl‐4PACz, and 2Cl‐5PACz, respectively (Figure S12, Supporting Information). Moreover, the HR‐XPS signals for C 1*s*, Cl 2*p*, N 1*s*, and P 2*p* from the SAMs (**Figure** **3**A–D), as well as In 3*d* from the ITO (Figure 3E), were compared for SAMs‐coated ITO samples. The detailed integrals for all those signals are summarized in Table S4 (Supporting Information). The relative coverage factor was determined by dividing the characteristic signal areas of C 1*s*, Cl 2*p*, N 1*s*, and P 2*p* by the number of corresponding atoms, and then normalizing these values to the signal area of In 3*d*_3/2_ (Figure 3F; Table S4, Supporting Information).^[^ ^16d^ ^]^ A relative coverage factor of 1.2 × 10^−2^, 7.7 × 10^−3^, 1.0 × 10^−2^, and 1.1 × 10^−2^ was found for 2Cl‐2PACz, 2Cl‐3PACz, 2Cl‐4PACz, and 2Cl‐5PACz‐modified ITO, respectively, based on their C 1*s* signals. More importantly, relative coverage factors derived from other characteristic elements (Cl 2*p*, N 1*s*, and P 2*p*) exhibited the same trend. The overall relative coverage factors decreased in the order: 2Cl‐2PACz > 2Cl‐5PACz > 2Cl‐4PACz > 2Cl‐3PACz. Moreover, the UV–vis spectra of spin‐coated SAMs on quartz glass, both before and after methanol washing, indicated that 2Cl‐3PACz has significantly lower absorbance than the other SAMs, suggesting it has the lowest coverage on quartz surfaces either in multilayer or monolayer structure (Figure S13, Supporting Information). This is consistent with the HR‐XPS result. In contrast, 2Cl‐2PACz, 2Cl‐4PACz, and 2Cl‐5PACz displayed similar absorbance intensities in the UV–vis spectra. We note that quantitative comparison of coverage differences among these three SAMs in monolayer from UV–vis spectra is challenging, as the weak signal intensity at ≈ 305 nm—≈0.036—is easily influenced by noise, which limits measurement accuracy. It is noteworthy that a better coverage is beneficial for charge transportation between active layers and electrodes.^[^ ^16a,d^ ^]^ We infer that differences in SAMs coverage may originate from variations in their molecular conformation and intermolecular interactions caused by their spacer lengths. This will be further discussed through single crystal data analyses.

### 2.4 Single Crystals Analyses of Intermolecular Interactions for SAMs

**Figure 4:**
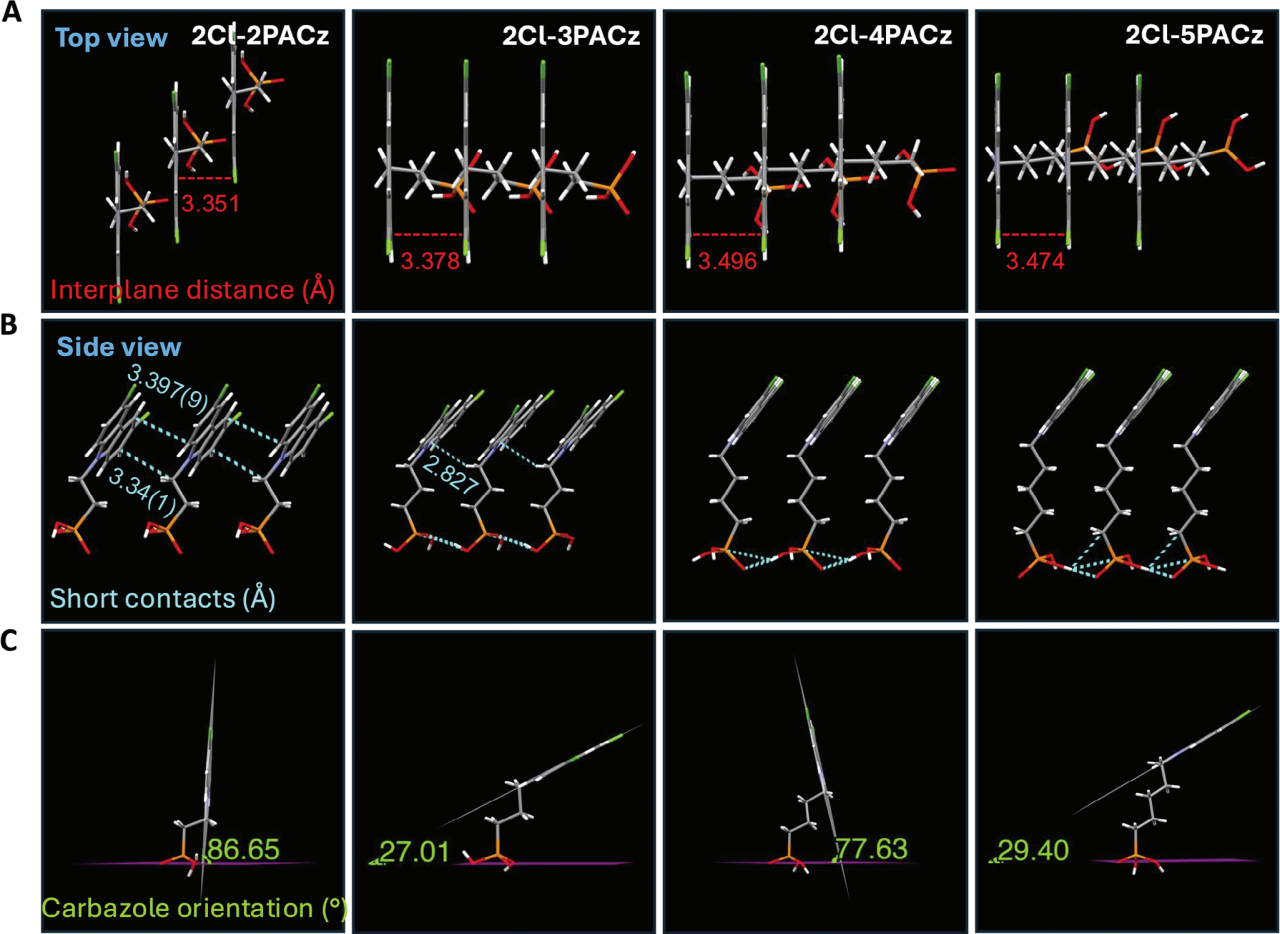
The molecular packing patterns in single crystals and single molecular conformation of 2Cl‐2PACz, 2Cl‐3PACz, 2Cl‐4PACz, and 2Cl‐5PACz. (A) The top view of the interplane distance is based on the carbazole planes. (B) The side view of the intermolecular short contacts within 4.0 Å. (C) Illustration of orientation of the carbazole relative to the horizontal plane defined by the three oxygen atoms of the anchor group, based on their single molecular conformations of SAMs. (CCDC No.: 2352347 for 2Cl‐2PACz, 2366230 for 2Cl‐3PACz, 2366232 for 2Cl‐4PACz and 2366233 for 2Cl‐5PACz).

To investigate the impact of the alkyl spacer length in SAMs on intermolecular interactions and molecular conformation, the direct method of X‐ray study of single crystal has been deeply applied. It could also help in understanding SAMs' packing behavior on ITO surfaces. All single crystals of the SAM materials investigated in this work were obtained from ethanol solutions. The vertical distances between carbazole planes, the short‐range interactions (Van der Waals forces within 4.0 Å) between adjacent SAMs, and the single molecular conformations of SAMs were thoroughly compared (**Figure** **4**). The vertical distances between carbazole planes gradually increased from 3.351 to 3.378, 3.496, and 3.474 Å for 2Cl‐2PACz, 2Cl‐3PACz, 2Cl‐4PACz, and 2Cl‐5PACz, respectively (Figure 4A). Additionally, 2Cl‐2PACz showed two types of short‐range interactions between adjacent SAMs at 3.34(1) Å and 3.397(9) Å, whereas 2Cl‐3PACz exhibited one type at 2.827 Å. However, 2Cl‐4PACz and 2Cl‐5PACz did not display any discernible short‐range interactions (Figure 4B). These results indicate that SAMs with shorter spacer lengths exhibit denser packing and stronger intermolecular interactions. This may be because SAMs with shorter spacer lengths (2Cl‐2PACz and 2Cl‐3PACz) showed more C···H/H···C interactions between the carbazole planes or between the carbazole planes and spacers. In contrast, SAMs with longer spacer lengths (2Cl‐4PACz and 2Cl‐5PACz) exhibited more H···H interactions between the methylene groups of adjacent SAMs, as demonstrated by Hirshfeld surface and fingerprint plots analysis (Figures S14 and S15 and Table S5, Supporting Information). These results clearly explain how spacer length affects intermolecular interactions and their packing in single crystals. Additionally, differential scanning calorimetry (DSC) tests of SAMs powder showed that 2Cl‐2PACz exhibited one small and two medium‐sized peaks during the first heating ramp, while other SAMs displayed only one sharp melting peak in the first cycle (Figure S16, Supporting Information). This also proves that the spacer length has a significant effect on SAMs aggregation. A longer spacer length primarily results in a single type of aggregation in the solid‐state due to increased interactions between spacers. In contrast, 2Cl‐2PACz exhibited a wider variety of aggregations due to its shorter spacer length, with less defined crystalline structures.

### 2.5 Molecular Conformation of SAMs

Furthermore, the molecular conformations of SAMs can also affect their packing density on the ITO surface, due to the bidentate or tridentate binding modes between the PA groups of SAMs and the hydroxyl groups on ITO after plasma treatment. To explore these effects, we compared molecular conformations by positioning the three oxygen atoms in a plane parallel to the ITO surface, assuming tridentate binding (Figure 4C). First, a clear odd‐even effect of spacer length on carbazole plane orientation was observed. It was then found that the orientations of the carbazole plane relative to the plane of the three oxygen atoms in PA group for 2Cl‐2PACz, 2Cl‐3PACz, 2Cl‐4PACz, and 2Cl‐5PACz were 86.65°, 27.01°, 77.63°, and 29.40°, respectively. This may influence the occupied area of single molecule, thereby influencing the surface coverage of ITO. Although the coverage factors for these four SAMs, obtained from HR‐XPS results, are based on their multilayer structures, we suspect that these values may indirectly reflect the coverage density of the first layer, given that the preparation conditions for all SAM films were identical. This inference is further supported by UV–vis spectra, which showed that 2Cl‐3PACz exhibited the lowest coverage in both multilayer and monolayer structure (Figure S13, Supporting Information). As a result, among the SAMs, 2Cl‐2PACz achieved the highest coverage due to its smaller molecular footprint and strong intermolecular interactions, as its carbazole group is nearly perpendicular to the ITO surface. In contrast, 2Cl‐3PACz displayed the lowest coverage, with the carbazole group more parallel to the ITO surface, resulting in a larger occupied area per molecule. For 2Cl‐4PACz and 2Cl‐5PACz, the longer spacer chains provided additional flexibility influencing the orientation of the carbazole headgroups and promoted H···H interactions among methylene groups, resulting in moderate and comparable coverage on the ITO surface. Hence, it can be inferred that the length of the alkyl spacer in SAMs significantly influences the molecular conformation and intermolecular interactions, leading to varying assembly patterns on ITO surfaces. Furthermore, based on the single‐molecule conformations of SAMs, we can derive precise height measurements ranging from 0.7 to 1.1 nm, which are valuable for estimating the thickness of SAMs monolayers or multilayers (Figure S17, Supporting Information).

### 2.6 Surface Morphology and Surface Energy

The surface morphologies of bare ITO and the ITO/SAMs were investigated by tapping‐mode atomic force microscopy (AFM). Height images showed that bare ITO and ITO/SAMs displayed similar root‐mean‐squared surface roughness (*R*_q_) of 2.36, 2.36, 2.43, 2.33, and 2.34 nm for ITO, ITO/2Cl‐2PACz, ITO/2Cl‐3PACz, ITO/2Cl‐4PACz, and ITO/5Cl‐2PACz (Figure S18, Supporting Information), respectively. These results indicate that the multilayer structures of different SAMs may be similar and have little effect on the surface morphology roughness of ITO glass. Moreover, considering that surface free energy of the HTLs can affect the microstructures of the active layer, contact‐angle measurements of bare ITO after plasma treatment, SAMs‐modified ITO, and active layer PM6:L8‐BO were performed, and their corresponding surface energy were calculated (Figure S19 and Tables S6 and S7, Supporting Information). The surface energies for bare ITO and active layer PM6:L8‐BO were of 88.33 and 28.90 mJ m^−2^, respectively. In comparison, those of 2Cl‐2PACz, 2Cl‐3PACz, 2Cl‐4PACz, and 2Cl‐5PACz were 38.42, 37.55, 38.03, and 38.09 mJ m^−2^, respectively. The surface energy of ITO/SAMs were very different from bare ITO, which confirms the presence of the SAMs on ITO and the reasonable coverage. Moreover, all SAMs exhibited a similar surface energy, which may have little effects on the active layer morphology.

### 2.7 Photovoltaic Performance of SAMs‐Based OSCs

**Figure 5:**
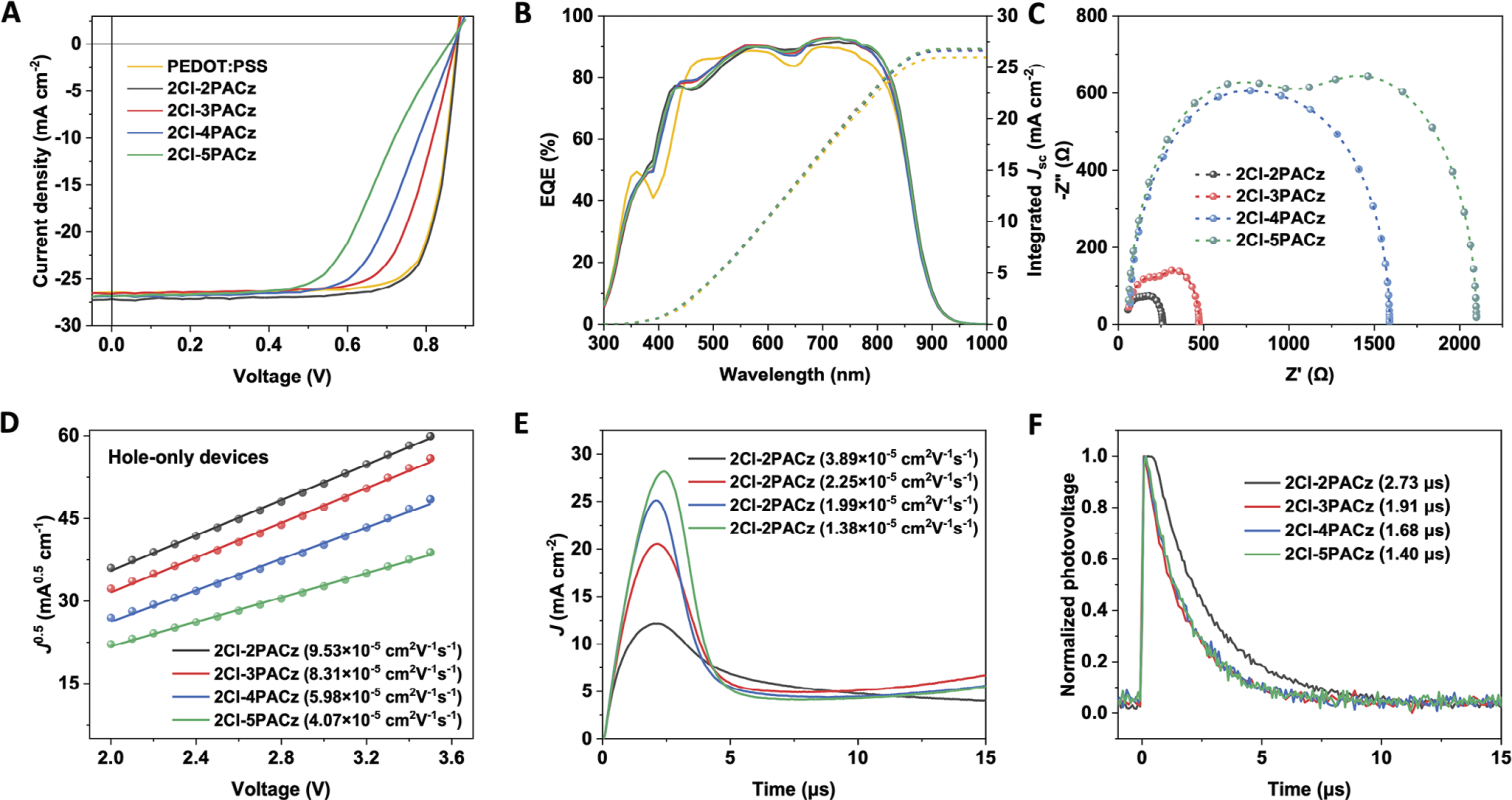
(A) The *J*–*V* characteristics with PM6:L8‐BO bulk heterojunction (BHJ) system. (B) EQE spectra. (C) EIS of OSC devices with 2Cl‐2PACz, 2Cl‐3PACz, 2Cl‐4PACz, and 2Cl‐5PACz HTLs. (D) SCLC of hole‐only devices with different HTLs. (E) Photo‐CELIV and (F) normalized TPV graphs of OSC devices with different HTLs.

Based on our encouraging findings, 2Cl‐2PACz, 2Cl‐3PACz, 2Cl‐4PACz, and 2Cl‐5PACz were applied as HTLs in OSCs to evaluate the alkyl spacer length effects of SAMs on PV performance. We fabricated OSC devices with a standard architecture of ITO/SAMs or PEDOT: PSS/PM6:L8‐BO/PNDIT‐F3N/Ag. **Figure** **5**A presents representative current–voltage (*J‐* *V*) curves for the devices with different ITO/SAMs or PEDOT: PSS anodes, with the key cell parameters summarized in **Table** **1**. The OSC devices based on ITO/2Cl‐2PACz anode exhibited a maximum PCE of 18.62% with a short‐circuit current density (*J*_sc_) of 27.32 mA cm^−2^, an open circuit voltage (*V*_oc_) of 0.880 V, and an FF of 77.44% under 1 sun illumination (AM 1.5G, 100 mW cm^−2^). As the alkyl spacer length increased, a gradual decrease in PCE, FF, and *V*_oc_ was observed from 2Cl‐3PACz to 2Cl‐5PACz. Specifically, PCE decreased from 16.61% and 15.21% to 13.27%, FF decreased from 71.10% and 64.57% to 57.38%, and *V*_oc_ decreased from 0.873 and 0.871 to 0.858 V, respectively. It was observed that the significant decrease in PCE is mainly attributed to the reduction in FF. The variation tendency is found accordant with the increase of series resistance (*R*_s_), from 31.1 to 93.0, 154.1, and 240.2 Ω for 2Cl‐2PACz, 2Cl‐3PACz, 2Cl‐4PACz, and 2Cl‐5PACz, respectively. Evidently, a longer spacer in SAMs leads to a higher hole injection barrier from the active layer to the electrode due to the increased hole tunneling distance of SAM layer.^[^ ^18^, ^31^ ^]^ Notably, in both monolayer and multilayer structures, the hole tunneling distance is expected to increase as the alkyl spacer lengthens from 2Cl‐2PACz to 2Cl‐5PACz, due to the increased molecular length.

**Table 1:** Photovoltaic parameters of OSCs based on PM6:L8‐BO BHJs with different SAMs as HTLs and PEDOT: PSS measured under illumination with AM 1.5G (100 mW cm^−2^).

HTLs	*V*_oc_ [V]	*J*_sc_ [mA cm^−2^]	*J*_cal_ ^a)^ [mA cm^−2^]	FF [%]	PCE^b)^ [%]	*R*_s_ [Ω]	*R*_ele, int, bhj_ [Ω]
PEDOT: PSS	0.878	26.54	25.96	78.75	18.36 (18.28 ± 0.01)	27.8	–
2Cl‐2PACz	0.880	27.32	26.72	77.44	18.62 (17.94 ± 0.39)	31.1	260.4
2Cl‐3PACz	0.873	26.76	26.68	71.10	16.61 (16.43 ± 0.25)	93.0	475.4
2Cl‐4PACz	0.871	27.03	26.60	64.57	15.21 (14.74 ± 0.44)	154.1	1585.4
2Cl‐5PACz	0.858	26.94	26.80	57.38	13.27 (12.70 ± 0.65)	240.2	2106.4

^a)^ The integral *J*_sc_ values were calculated from the EQE curves;

^b)^ The average PCE values in brackets were obtained from 7 to 15 devices.

Additionally, similar *J*_sc_ ranging from 26.76 to 27.32 mA cm^−2^ were observed for all ITO/SAMs anodes‐based OSCs. The integrated photocurrent density (*J*_cal_) was obtained from external quantum efficiency (EQE) and was found to match the *J*_sc_ within ±3% (Figure 5B and Table 1). Moreover, the conventional PEDOT: PSS‐based OSC, used as a benchmark, shows a slightly lower PCE of 18.36% and a *J*_sc_ of 26.54 mA cm^−2^. This is due to an enhanced EQE observed between 370 to 420 nm and 550 to 860 nm for SAMs‐based OSCs compared to PEDOT: PSS‐based ones.

### 2.8 Device Impedance and Carrier Transportation

To further clarify the reasons for the different PV performances of the ITO/SAMs‐based OSCs, we examined the electrochemical impedance, carrier mobility, and carrier lifetime properties of the ITO/SAMs‐based devices. First, we performed electrochemical impedance spectroscopy (EIS) measurements to study the electrical properties of the OSC devices.^[^ ^16^, ^32^ ^]^ Figure 5C presents the Nyquist plots obtained for various OSC cells, with the corresponding fitting results detailed in Table 1 and Figure S20 and Table S8 (Supporting Information). Remarkably, the device resistance, encompassing the electrode resistance (*R*_ele_), interface resistance (*R*_int_), and BHJ layer resistances (*R*_bhj_), exhibited a remarkable increment from 260.4 to 475.4, 1585.4, and ultimately reaching 2106.4 Ω for devices from 2Cl‐2PACz to 2Cl‐5PACz. This increase in device resistance is concomitant with the changing trend of *R*_st_, further proving a direct correlation between the increased device resistance and reduced photovoltaic performances. Second, we fabricated hole‐only devices with an architecture of ITO/SAMs/active layer (100 nm)/MoO_3_ (10 nm)/Ag to employ to investigate the hole mobility (*μ*_h_) through space‐charge limited current (SCLC) measurements (Figure 5D).^[^ ^16^, ^22^ ^]^ The *μ*_h_ in device based on 2Cl‐2PACz (9.53 × 10^−5^ cm^2^ V^−1^ s^−1^) was higher than those based on 2Cl‐3PACz (8.31 × 10^−5^ cm^2^ V^−1^ s^−1^), 2Cl‐4PACz (5.98 × 10^−5^ cm^2^ V^−1^ s^−1^) and 2Cl‐5PACz (4.07 × 10^−5^ cm^2^ V^−1^ s^−1^), indicating that shorter spacers possess leads to more efficient hole extraction/transport. To further verify this hypothesis, the mobility (*μ*) of the carrier in OSC devices was investigated via photo‐induced charge‐carrier extraction in a linearly increasing voltage (photo‐CELIV) technique (Figure 5E).^[^ ^16^, ^18^ ^]^ The *μ* for 2Cl‐2PACz‐based OSC is the highest among all devices with different ITO/SAMs anodes, and the variation trend also falls in agreement with the SCLC results. Furthermore, the transient photovoltage (TPV) measurements were also tested to obtain the charge carrier lifetime (τ) (Figure 5F).^[^ ^7^, ^16^, ^18^ ^]^

As expected, the τ for ITO/2Cl‐2PACz cells (2.73 µs) was longer than those of the devices based on 2Cl‐3PACz (1.91 µs), 2Cl‐4PACz (1.68 µs), and 2Cl‐5PACz (1.40 µs). All these results suggest that ITO/2Cl‐2PACz with the shorter alkyl spacer possesses lower resistance, faster carrier transfer rate, and longer carrier lifetime, thereby enhancing the overall device performance. This could be attributed to its shorter hole tunneling distance and barrier,^[^ ^27^, ^31^ ^]^ higher WF, as well as the denser packing on ITO surfaces.^[^ ^16a,d^ ^]^ Additionally, the capacitance–voltage (*C–V*) in dark condition of these four devices were measured to obtain the build‐in potential (*V*_bi_) and the related Mott Schottky curves are recorded (Figure S21, Supporting Information). A gradually decreased *V*_bi_ was observed from 0.95, 0.94, 0.90, to 0.89 V for 2Cl‐2PACz to 2Cl‐5PACz, respectively, which can contribute the decreased charge extraction and *V*_oc_.^[^ ^28^ ^]^

### 2.9 Generality and Impact of HTL Rinsing‐Treatment on Photovoltaic Performance

**Figure 6:**
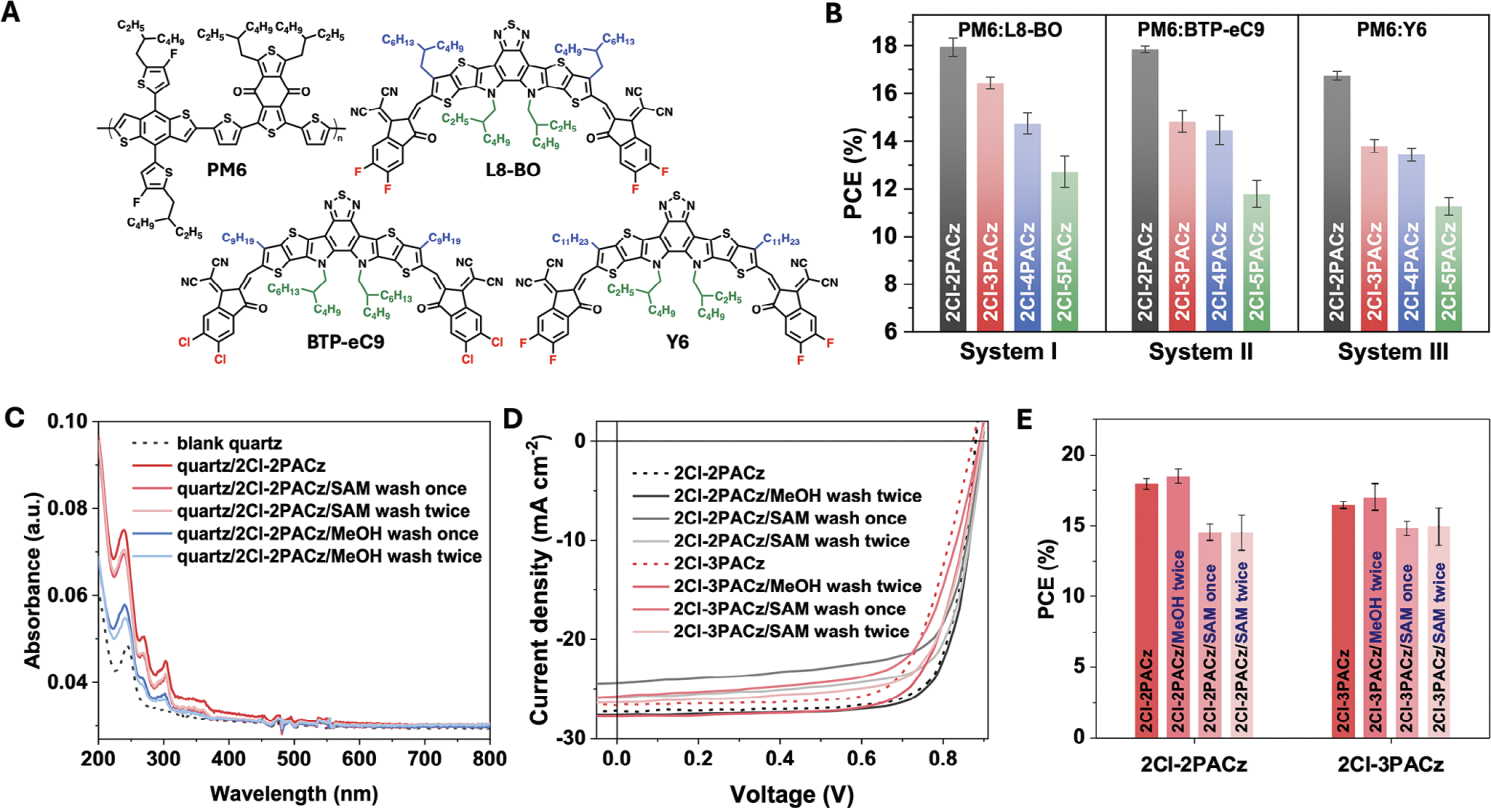
(A) Molecular structures of PM6, L8‐BO, BTP‐eC9 and Y6. (B) Comparison of PCEs from OSCs based on several active layers (PM6:L8‐BO, PM6:BTP‐eC9, and PM6:Y6) and with different HTLs materials. The ITO/SAMs layer was prepared without any rinsing treatment. (C) UV–vis absorption spectra of bare quartz glass and quartz glass spin‐coated with 2Cl‐2PACz, both before and after washing with methanol or the same 2Cl‐2PACz solutions. (D) *J‐* *V* characteristics of OSCs with ITO/SAMs under different rinsing treatments. (E) Comparison of PCEs of OSCs with varying rinsing treatments for the ITO/SAMs layer.

To evaluate the generality of the effects of alkyl spacer length on PV performance, 2Cl‐2PACz to 2Cl‐5PACz were tested on two additional systems based on PM6:BTP‐eC9 and PM6:Y6 BHJ active layers (**Figure** **6**A,B; Figure S22 and Table S9, Supporting Information). In both cases, the efficiencies decreased in a similar trend as for PM6:L8‐BO upon increasing the spacer length (2Cl‐2PACz > 2Cl‐3PACz > 2Cl‐4PACz > 2Cl‐5PACz). This confirms that a shorter alkyl spacer in SAMs generally enhances PV performance in OSCs, and make our observations applied to the field of OSCs in a more general way.

Moreover, we noticed that several research groups have employed different post‐treatment procedures for spin‐coated ITO/SAMs layers. These treatments include no rinsing step,^[^ ^7^, ^24^ ^]^ rinsing with pure solvents (e.g., methanol, ethanol, and isopropanol),^[^ ^18^, ^28^ ^]^ and rinsing with the same SAMs solution.^[^ ^16^, ^18^ ^]^ Herein, we chose 2Cl‐2PACz and 2Cl‐3PACz as the molecules of interest to explore how the rinsing step affects both the film thickness and PV performance. ITO/SAMs anodes were rinsed with methanol or the same SAMs solution (0.3 mg mL^−1^, 150 µL, 6000 rpm), once and twice, to compare the UV–vis spectra and PV performance to the devices without rinsing (Figure 6C–E). The key cell parameters are summarized in Table S10 (Supporting Information). First, it was observed that rinsing with SAMs solution once or twice slightly decreased the absorbance intensity of the SAMs‐modified quartz glass from UV–vis spectra (Figure 6C). This suggests that rinsing with SAMs solution may remove some SAM molecules or influence the molecular packing and orientation of the upper layers. In contrast, rinsing with methanol once or twice significantly reduced the absorbance intensity. Only the first monolayer was retained due to the strong covalent bonding between the PA groups and hydroxyl groups on the ITO surface, while the additional layers were washed out. Interestingly, rinsing the ITO/SAMs layer with methanol twice significantly enhanced the PCE of OSCs based on ITO/2Cl‐2PACz and ITO/2Cl‐3PACz to 18.95% and 17.85%, respectively (Figure 6D,E). These findings suggest that methanol rinsing can effectively remove the layers in excess and while retaining a monolayer, thereby further reducing the hole tunneling distance and barrier compared to multilayer structures, leading to enhanced PCEs. In contrast, rinsing the ITO/SAMs layer with SAMs solution once or twice results in decreased FF and PCE, potentially due to the morphological changes in the upper layers.

### 2.10 Summary of the Effect of Spacer Length of SAMs on Molecular Conformation and Photovoltaic Performance

**Figure 7:**
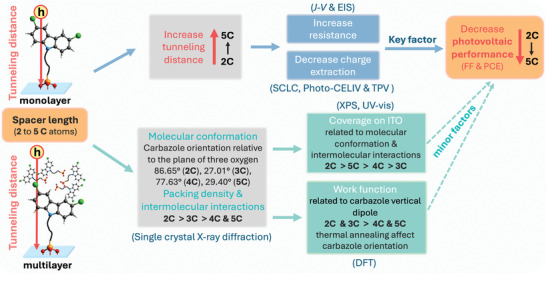
Illustration of the effect of spacer length of SAMs on molecular conformations, self‐assembling on ITO surface, and photovoltaic properties.

From the above discussion, an illustrative graph was used to reveal the effect of spacer length in SAMs on molecular conformation, intermolecular interaction, coverage on the ITO, WF of ITO, and the resulting PV performance of OSC devices (**Figure** **7**). As the spacer length increases, the hole tunneling distance and barrier are correspondingly increased in either monolayer or multilayer structure of SAMs layer, leading to an increase in both series resistance and overall device resistance. The increased tunneling distance and barrier decrease the carrier transportation rates and hinders charge extraction from the active layer to the anode, thereby reducing the FF and PCE of OSC devices. Additionally, the spacer length of SAMs affects the molecular conformation and intermolecular interactions, which significantly influences their coverage on the ITO surface. Considering SAMs adopt a tridentate binding way of PA anchoring group on the ITO surface, the angle between the carbazole plane and the plane formed by the three oxygen atoms in the PA group will greatly affect the surface occupation on ITO for each SAM molecule. 2Cl‐2PACz, with a large angle, together with the stronger intermolecular interactions, achieves the densest coverage on the ITO surface, while 2Cl‐3PACz, with a smaller angle, shows the least coverage. For 2Cl‐4PACz and 2Cl‐5PACz, the longer spacer chains provide additional flexibility influencing the orientation of the carbazole headgroups and promote H···H interactions among methylene groups, resulting in moderate and comparable coverage on the ITO surface. DFT simulations demonstrated that the WF of SAMs‐modified ITO is related to the vertical component of the molecular dipole moment. However, UPS results were not fully aligned with DFT simulations. This discrepancy suggests that the WF of SAMs‐modified ITO is likely influenced by combined factors of the original molecular conformation, the self‐assembling process, and thermal annealing process. Based on the above observations, we can conclude that in OSC devices based on SAMs, the primary factor affecting photovoltaic performance is the variation in spacer length, which alters the hole tunneling distance and barrier. In comparison, factors such as coverage and WF appear to be of secondary importance.

## 3 Conclusion

In this study, we systematically investigated the impact of alkyl spacer length in carbazole‐based SAMs (2Cl‐2PACz, 2Cl‐3PACz, 2Cl‐4PACz, and 2Cl‐5PACz) on molecular conformation, intermolecular interactions, packing density on ITO surfaces, WF of SAMs‐modified ITO, and photovoltaic performance of OSCs. Single crystal analysis showed that SAMs with shorter spacer led to denser packing and stronger intermolecular interactions, while longer spacers introduced additional H···H interactions between spacer chains. Additionally, the orientations of the carbazole plane relative to the plane of the three oxygen atoms in phosphate group were of 86.65°, 27.01°, 77.63°, and 29.40°, for 2Cl‐2PACz, 2Cl‐3PACz 2Cl‐4PACz, and 2Cl‐5PACz, respectively, which likely affected the molecular footprint and coverage on ITO substrate. This led to the best coverage of 2Cl‐2PACz on ITO, and the least coverage of 2Cl‐3PACz. More importantly, OSCs based on 2Cl‐2PACz achieved the highest PCE (18.62%) and FF (77.44%), while both photovoltaic parameters gradually decreased with increasing alkyl spacer length. A set of comprehensive characterizations revealed that 2Cl‐2PACz‐based devices exhibited lower series resistance and device resistance, faster hole/carrier mobility, and longer carrier lifetime, mainly attributed to shorter hole tunneling distance and lower tunneling barrier. The higher work function and denser coverage of 2Cl‐2PACz on ITO surfaces may also promote photovoltaic performance but are less important factors here as compared to the shorter hole tunneling distance. Moreover, the experiments from UV–vis spectra indicated that the spin‐coating of SAM materials onto ITO untreated or rinsing with SAMs solution can only form a multilayer structure, while rinsing with methanol yield an actual monolayer. The rinsing with methanol further enhanced the PCE to 18.95% for OSCs with ITO/2Cl‐2PACz anodes. Overall, this research sheds light on our understanding of how alkyl spacer length in SAMs affects molecular conformation and packing, coverage and arrangement on ITO surfaces, and the photovoltaic performance of OSCs. Moreover, it provides valuable insights for the design of SAM materials for high‐performance OSCs and other related devices.

[CCDC 2352347, 2366230, 2366232, and 2366233 contain the supplementary crystallographic data for this paper. These data can be obtained free of charge from The Cambridge Crystallographic Data Centre via www.ccdc.cam.ac.uk/data_request/cif.]

## Conflict of Interest

The authors declare no conflict of interest.

## Supporting information



Supporting Information

## Data Availability

The data that support the findings of this study are available in the supplementary material of this article.
