# Supplementary material for: Effects of Alkyl Spacer Length in Carbazole‐Based Self‐Assembled Monolayer Materials on Molecular Conformation and Organic Solar Cell Performance
Source: Adv Sci (Weinh). 2024 Dec 4;12(4):2410277. doi: 10.1002/advs.202410277 (PMC11775572; doi:10.1002/advs.202410277)
Supplement: Supplementary file 1 — Supporting Information [file ADVS-12-2410277-s001.docx]

Supporting Information

Effects of Alkyl Spacer Length in Carbazole-based Self-Assembled Monolayer Materials on Molecular Conformation and Organic Solar Cell Performance

Qiaonan Chen, Kangbo Sun, Leandro R. Franco, Jingnan Wu, Lars Öhrström, Xianjie Liu, Maureen Gumbo, Mailde S. Ozório, C. Moyses Araujo, Guangye Zhang, André Johansson, Ellen Moons, Mats Fahlman, Donghong Yu, Yufei Wang*, and Ergang Wang*

**Table of Contents**

1. Materials and methods
2. Synthesis and characterization
3. Supporting Figures S1 to S22 & Tables S1 to S11
4. Nuclear magnetic resonance spectra Figures S23 to S48
5. Supplemental references
6. **Materials and methods**

**Materials:**

Carbazole, N-chlorosuccinimide (NCS), 1,2-dibromoethane, 1,4-dibromobutane, 1,5-dibromopentane, tetrabutylammonium bromide, triethyl phosphite and bromotrimethylsilane were purchased from Sigma Aldrich. 1,3-Dibromopropane was bought from TCI. PM6 was purchased from Solarmer Materials In. L8-BO, BTP-eC9, Y6 and PNDIT-F3N were acquired from eFlexPV Limited. PEDOT:PSS (Clevios PVP 4083) was purchased from Heraeus Inc. All the other reagents and chemicals were purchased from Sigma Aldrich or Aladdin and used as received.

**Full names of the abbreviations for materials in the main manuscript**:

Poly[(9,9-bis(3'-((N,N-dimethyl)-N-ethylammonium)-propyl)-2,7-fluorene)-alt-2,7-(9,9-dioctylfluorene)]dibromide (PFN-Br), Poly[[2,7-bis(2-ethylhexyl)-1,2,3,6,7,8-hexahydro-1,3,6,8-tetraoxobenzo[lmn][3,8]phenanthroline-4,9-diyl]-2,5-thiophenediyl[9,9-bis[3-(dimethylamino)propyl]-9H-fluorene-2,7-diyl]-2,5-thiophenediyl] (PNDIT-F3N), 2,9-Bis[3-(dimethyloxidoamino)propyl]anthra[2,1,9-def:6,5,10-d'e'f']diisoquinoline-1,3,8,10(2H,9H)-tetrone (PDINO), 2,9- bis(3-(dibutylamino)propyl)perylene diimide (PDINB), N,N'-Bis{3-[3-(dimethylamino)propylamino]propyl}perylene-3,4,9,10-tetracarboxylic diimide (PDINN), Poly[[4,8-bis[5-(2-ethylhexyl)-4-fluoro-2-thienyl]benzo[1,2-b:4,5-b']dithiophene-2,6-diyl]-2,5-thiophenediyl[5,7-bis(2-ethylhexyl)-4,8-dioxo-4H,8H-benzo[1,2-c:4,5-c']dithiophene-1,3-diyl]-2,5-thiophenediyl] (PM6), 2,2'-((2Z,2'Z)-((3,9-bis(2-butyloctyl)-12,13-bis(2-ethylhexyl)-12,13-dihydro-[1,2,5]thiadiazolo[3,4-e]thieno[2'',3'':4',5']thieno[2',3':4,5]pyrrolo[3,2-g]thieno[2',3':4,5]thieno[3,2-b]indole-2,10-diyl)bis(methanylylidene))bis(5,6-difluoro-3-oxo-2,3-dihydro-1H-indene-2,1-diylidene))dimalononitrile (L8-BO), 2,2'- [[12,13-Bis(2-butyloctyl)-12,13-dihydro-3,9-dinonylbisthieno[2'',3'':4',5']thieno[2',3':4,5]pyrrolo[3,2-e:2',3'-g][2,1,3]benzothiadiazole-2,10-diyl]bis[methylidyne(5,6-chloro-3-oxo-1H-indene-2,1(3H)-diylidene)]]bis[propanedinitrile] (BTP-eC9), 2,2'-((2Z,2'Z)-((12,13-Bis(2-ethylhexyl)-3,9-diundecyl-12,13-dihydro-[1,2,5]thiadiazolo[3,4-e]thieno-[2'',3'':4',5']thieno[2',3':4,5]pyrrolo[3,2-g]thieno-[2',3':4,5]thieno[3,2-b]indole-2,10-diyl)bis(methanylylidene))-bis(5,6-difluoro-3-oxo-2,3-dihydro-1H-indene-2,1-diylidene))dimalononitrile (Y6).

**Methods:**

^1^H and ^13^C NMR spectra were recorded on 600 MHz Oxford magnet equipped with Bruker NEO console and QCIP ^1^H/^19^F/^31^P/^13^C/^15^N cold probe, with residual solvent signals as internal reference (^1^H NMR: DMSO-*d*_6_, 2.50 ppm; ^13^C{^1^H} NMR: DMSO-*d*_6_, 39.52 ppm). The following abbreviations were used to describe peak patterns when appropriate: br = broad signal, s = singlet, d = doublet, t = triplet, dd = doublet of doublets, m = multiplet. The ultraviolet-visible (UV-vis) absorption spectra were recorded on a PerkinElmer lambda 1050 UV/vis/NIR spectrometer. Thermogravimetric analysis (TGA) was conducted by a Mettler Toledo TGA/DSC 3+ STAR System instrument under nitrogen atmosphere at a heating rate of 10 ℃ min^-1^. Differential scanning calorimetry (DSC) measurement was performed by using DSC 250 (TA Instruments). Ultraviolet photoelectron spectroscopy (UPS) measurements were performed by a Thermo Fisher Scientific K-ALPHA+ system with He I (21.22 eV) as the excitation source. X-ray photoelectron spectroscopy (XPS) (Thermo Scientific K-Alpha) for the analysis of material element composition is produced by Thermo Fisher Scientific. The characterization of single crystals was conducted with Single Crystal X-ray Diffraction (SCXRD) instrument (XtaLAB Synergy-R from Rigaku). A NTEGRA AFM (NT-MDT) instrument was used with Tap300Al-G tips (BudgetSensors®) with 200-400 kHz resonant frequency, 40 N/m force constant and 10 nm tip radius. The tapping mode AFM images were recorded. Post-processing of data includes levelling using a mean plane subtraction, followed by row alignment using a second order polynomial and offsetting to zero at the lowest measured height.

**OSCs Fabrication:**

The normal type OSCs (indium tin oxide (ITO)/SAMs or poly(3,4-ethylenedioxy thiophene):polystyrene sulfonic acid (PEDOT: PSS, AI4083 from Heraeus)/active layer/interlayer/Ag) were fabricated by following processes. Poly[[2,7-bis(2-ethylhexyl)-1,2,3,6,7,8-hexahydro-1,3,6,8-tetraoxobenzo[lmn][3,8]phenanthroline-4,9-diyl]-2,5-thiophenediyl[9,9-bis[3'((N,N-dimethyl)-N-ethylammonium)]-propyl]-9H-fluorene-2,7-diyl]-2,5-thiophenediyl] (PNDIT-F3N) was used as interfacial layer. ITO-coated glass substrates were treated with ultrasonication by detergent, DI-water, acetone and isopropyl alcohol. The cleaned substrates were dried for more than 1 hr at 80 ℃. A plasma treatment was proceeded for 8 min before spin-casting SAMs or PEDOT:PSS solution. 150 µL of SAMs solution (0.3 mg mL^-1^, dissloved in methanol) was spin-casted at 3000 rpm for 30 s before staying on the ITO glass for 1 minute, then thermally annealed at 75 ℃ for 5 min in glove box filled with nitrogen gas. This treatment for the ITO/SAMs layer was utilized in most of the characterizations in this study. Only for the comparison of the effects of rinsing treatments on the ITO/SAMs layer, 150 µL of methanol or the same SAM solution was spin-coated onto the top of the ITO/SAMs layer at 6000 rpm for 30 seconds, either once or twice. The PEDOT: PSS solution was spin-casted at 4000 rpm for 30 s, then thermally annealed at 150 ℃ for 15 min in ambient condition. Then, the samples were carried to a N_2_-filled glovebox. Next, the PM6:L8-BO blend solutions with optimal concentration (17.6 mg mL^−1^) and *P*_D_:*P*_A_ ratio (1:1.2) were prepared in chloroform solvent. The solutions were stirred for at least 1 hr at 55 ℃, and the 0.25% vol DIO was added before spin-coating. Then, solutions were spin-casted onto the PEDOT:PSS-coated or SAM-coated ITO substrates at 3000 rpm for 30 s, and the films were thermally annealed at 100 ℃ for 5 min. After that, the samples were dried at a high-vacuum chamber for 2 hrs. Then, a methanol with 0.5% vol acetic acid blend solution of PNDIT-F3N at a concentration of 0.5 mg mL^-1^ was spin-coated onto the active layer films with 2000 rpm for 30 s. Finally, Ag electrode (120 nm) was deposited by thermal evaporation in an evaporation chamber, under a high vacuum (~10^-6^ Torr) condition. The photoactive area of the OSC devices is 0.072 cm^2^, measured from the optical microscopy. The mask area for measurement is 0.0395 cm^2^.

**OSCs measurements:**

The current density-voltage (*J-V*) curves of all encapsulated devices were measured using a Keithley 2400 Source Meter under AM 1.5G (100 mW cm^-2^) using an Enlitech solar simulator. The light intensity was calibrated using a standard Si diode with KG5 filter to bring spectral mismatch to unity. Optical microscope (Olympus BX51) was used to define the device area (7.2 mm²). EQEs were measured using an Enlitech QES EQE system equipped with a standard Si diode. Monochromatic light was generated from a Enlitech 300W lamp source. In TPV measurements, the devices were placed under background light bias enabled by a focused Quartz Tungsten-Halogen Lamp with an intensity like working devices, i.e., the device voltage matches the open-circuit voltage under solar illumination conditions. Photo-excitations were generated with an 8 ns pulses from a laser system (Oriental Spectra, NLD520). The wavelength for the excitation was tuned to 518 nm with a spectral width of 3 nm. A digital oscilloscope was used to acquire the TPV signal at the open-circuit condition. The impedance spectrum, capacitance-frequency and Photo-CELIV measurements were performed using commercially available Paios system (FLUXiM AG). The contact angle images of films were obtained from optical contact angle measuring and contour analysis systems (OCA 15EC).

**SCLC Measurement:**

The hole-only devices for the SCLC measurement have device architectures of ITO/ SAMs/active layer (100 nm)/MoO_3_ (10 nm)/Ag. The semiconducting films were spin-casted in the N_2_-filled glovebox, and the blend films were prepared in the same condition as that for OSCs fabrication. Mott-Gurney equation was used to fit the *J*-*V* characteristics:

$J_{SCLC}\text{ }=\frac{9}{8}\varepsilon_{0}\varepsilon_{r}\mu(V^{2}/L^{3})$.

($\varepsilon_{0}$ = the free-space permittivity, $\varepsilon_{r}$ = the dielectric constant of the semiconductor, $\mu$ = the mobility, *V* = the applied voltage, and *L* = the thickness of the active layer.)

**Surface energy calculation of film**

The solid surface energy using Owen Method was based on the following model:

$\gamma_{s}=\gamma_{S}^{D}+\gamma_{S}^{P}$ (1)

$\gamma_{l}=\gamma_{l}^{D}+\gamma_{l}^{P}$ (2)

where $\gamma_{s}$ stands for the surface of the solid, which consists of the polarity force $\gamma_{S}^{P}$ and dispersion force $\gamma_{S}^{D}$, and $\gamma_{l}$ refers to the surface energy of the liquid with the corresponding polarity force $\gamma_{l}^{P}$ and dispersion force $\gamma_{l}^{D}$. According to the following equation, the $\gamma_{S}^{P}$ and $\gamma_{S}^{D}$ of measured film can be acquired.

$\gamma_{l}\left( 1+cos\theta\right)=2\left( \gamma_{S}^{D}\gamma_{l}^{D} \right)^{1/2}+2{(\gamma_{S}^{P}\gamma_{l}^{P})}^{1/2}$ (3)

In this work, the water (H_2_O) and ethylene glycol (EG) are used as the testing liquids to extract the $\gamma_{S}^{P}$ and $\gamma_{S}^{D}$. The related parameters of H_2_O and EG are listed in **Table S9**.

1. **Synthesis and characterization**

**Synthesis of SAMs**

**Scheme S1.** Synthesis route of SAMs, 2Cl-2PACz, 2Cl-3PACz, 2Cl-4PACz and 2Cl-5PACz.

**2**^[1]^, **5**^[2]^ and 2Cl-2PACz^[3]^ were synthesized according to the references.

**3,6-dichloro-9*H*-carbazole (2)** In a 250 mL dry two-necked round-bottom flask, 9*H*-carbazolethiophene (**1**, 1 eq, 12.0 g, 71.8 mmol) and 50 mL anhydrous DMF were mixed under nitrogen protection, followed by addition of the solution of N-chlorosuccinimide (NCS, 2.04 eq, 19.6 g, 146.7 mmol) in 100 mL anhydrous DMF dropwise. The reaction mixture was stirred at 60 ℃ for 4 hrs and then removed under reduced pressure. The target compound **2** was finally obtained through recrystallization with chloroform for three times to give the white needle solid state compound (16.95 g, yield 69%). ^1^H NMR (600 MHz, DMSO-*d*_6_, TMS), δ (ppm): 11.57 (s, 1H), 8.29 (d, *J* = 2.2 Hz, 2H), 7.52 (d, *J* = 9.2 Hz, 2H), 7.42 (dd, *J* = 8.6, 2.1 Hz, 2H). ^13^C NMR (150 MHz, DMSO-*d*_6_, TMS), δ (ppm): 138.7, 126.1, 123.2, 122.9, 120.3, 112.8.

**9-(2-bromoethyl)-3,6-dichloro-9*H*-carbazole (3a)** 3,6-dichloro-9*H*-carbazole (**2**, 1 eq, 1.67 g, 7.1 mmol) was dissolved in 1,2-dibromoethane (16 eq, 19.7 g, 113 mmol), tetrabutylammonium bromide (0.14 eq, 319 mg, 1 mmol) and 50% KOH aqueous solution (4.6 eq, 1.82 g, 32.4 mmol) were added subsequently. Reaction was stirred at 70 °C overnight. TLC analysis reveals that starting material **2** remains present within the reaction system. Extra tetrabutylammonium bromide (350 mg) and 50% KOH aqueous solution (2.0 g) was added. The reaction was run for another 24 hrs. After completion of the reaction, extraction was done with chloroform. The organic layer was washed with brine for three times, dried over anhydrous Na_2_SO_4_, and then the solvent was distilled off under reduced pressure. The crude product was purified by column chromatography (silica gel: 70 to 230 mesh; eluent: petroleum ether first to remove residual **2**, then petroleum ether: chloroform 3:1 v:v) to give **3a** as a white solid state compound (0.92 g, yield 38%). ^1^H NMR (600 MHz, DMSO-*d*_6_, TMS), δ (ppm): 8.34 (d, *J* = 2.1 Hz, 2H), 7.73 (d, *J* = 8.7 Hz, 2H), 7.50 (dd, *J* = 8.7, 2.0 Hz, 2H), 4.87 (t, *J* = 6.2 Hz, 2H),3.90 (t, *J* = 6.2 Hz, 2H). ^13^C NMR (150 MHz, DMSO-*d*_6_, TMS), δ (ppm): 139.0, 126.3, 123.9, 122.6, 120.4, 111.7, 44.1, 31.4.

**9-(4-bromobutyl)-3,6-dichloro-9*H*-carbazole (3b)** The same prepared procedure for synthesizing **3a**. The reaction was run 24 hrs. The crude product was purified by column chromatography (silica gel: 70 to 230 mesh; eluent: petroleum ether first to remove residual **2**, then petroleum ether: chloroform 8:1 v:v) to give **3b** as a white solid state compound (2.53 g, yield 78%). ^1^H NMR (600 MHz, DMSO-*d*_6_, TMS), δ (ppm): 8.32 (d, *J* = 2.1 Hz, 2H), 7.68 (d, *J* = 8.7 Hz, 2H), 7.49 (dd, *J* = 8.7, 2.2 Hz, 2H), 4.43 (t, *J* = 6.9 Hz, 2H), 3.51 (t, *J* = 6.5 Hz, 2H), 1.88-1.76 (m, 4H). ^13^C NMR (150 MHz, DMSO-*d*_6_, TMS), δ (ppm): 138.9, 126.2, 123.5, 122.4, 120.4, 111.2, 41.7, 34.5, 29.7, 27.1.

**9-(5-bromopentyl)-3,6-dichloro-9*H*-carbazole (3c)** The same prepared procedure for synthesizing **3a**. The reaction was run 24 hrs. The crude product was purified by column chromatography (silica gel: 300 to 400 mesh; eluent: petroleum ether first to remove residual **2**, then petroleum ether: chloroform 6:1 v:v) to give **3c** as a white solid state compound (1.02 g, yield 45%). ^1^H NMR (600 MHz, DMSO-*d*_6_, TMS), δ (ppm): 8.33 (d, *J* = 2.1 Hz, 2H), 7.67 (d, *J* = 8.7 Hz, 2H), 7.49 (dd, *J* = 8.7, 2.1 Hz, 2H), 4.41 (t, *J* = 7.1 Hz, 2H), 3.46 (t, *J* = 6.7 Hz, 2H), 1.82-1.74 (m, 4H),1.41-1.36 (m, 2H). ^13^C NMR (150 MHz, DMSO-*d*_6_, TMS), δ (ppm): 138.9, 126.2, 123.5, 122.4, 120.4, 111.3, 42.4, 34.9, 31.9, 27.6, 25.0.

**(2-(3,6-dichloro-9*H*-carbazol-9-yl)ethyl)phosphonate (4a)** In a 100 mL dry round-bottom flask, 9-(2-bromoethyl)-3,6-dichloro-9*H*-carbazole (**3a**, 1 eq, 1.07 g, 3.0 mmol) was dissolved in triethyl phosphite (20 eq, 15.0 g, 90.0 mmol). The reaction mixture was stirred at 150 ℃ for 2 days. After completion of the reaction, some white solids were found precipitated out from the reaction solution. After cooling down, petroleum ether was added into the system and then put in the fridge. The target compound **2** was finally obtained through filtering and washed with petroleum ether as a white solid compound (0.929 g, yield 74%).^1^H NMR (600 MHz, DMSO-*d*_6_, TMS), δ (ppm): 8.33 (d, *J* = 2.1 Hz, 2H), 7.62 (d, *J* = 8.7 Hz, 2H), 7.51 (dd, *J* = 8.7, 2.2 Hz, 2H), 4.61-4.57 (m, 2H), 3.87-3.82 (m, 4H), 2.29 (dt, *J* = 18.0, 7.1 Hz, 2H), 1.04 (t, *J* = 7.0 Hz, 6H). ^13^C NMR (150 MHz, DMSO-*d*_6_, TMS), δ (ppm): 138.6, 126.2, 123.7, 122.7, 120.4, 111.4, 61.1, 37.0, 24.5, 23.6, 15.9.

**(4-(3,6-dichloro-9*H*-carbazol-9-yl)butyl)phosphonate (4b)** The same prepared procedure for synthesizing **4a**. However, the work-up procedure is different. The reaction was run for 24 hrs. After completion of the reaction, the triethyl phosphite was removed under reduced pressure. The crude product was purified by column chromatography (silica gel: 70 to 230 mesh; eluent: acetone: petroleum ether 1:4 v:v) to give **4b** as a white solid state compound (1.7 g, yield 64%). ^1^H NMR (600 MHz, DMSO-*d*_6_, TMS), δ (ppm): 8.32 (d, *J* = 2.1 Hz, 2H), 7.69 (d, *J* = 8.7 Hz, 2H), 7.48 (dd, *J* = 8.7, 2.2 Hz, 2H), 4.41 (t, *J* = 7.1 Hz, 2H), 3.93 – 3.84 (m, 4H), 1.84 – 1.79 (m, 2H), 1.72 (ddd, *J* = 17.9, 9.1, 6.8 Hz, 2H), 1.51 – 1.41 (m, 2H), 1.13 (t, *J* = 7.0 Hz, 7H). ^13^C NMR (150 MHz, DMSO-*d*_6_, TMS), δ (ppm): 139.0, 126.2, 123.5, 122.4, 120.4, 111.3, 60.8, 42.1, 29.1, 24.6, 23.7, 19.6, 16.2.

**(5-(3,6-dichloro-9H-carbazol-9-yl)pentyl)phosphonate (4c)** The same prepared procedure for synthesizing **4a**. The reaction was run for 24 hrs. The crude product was purified by column chromatography (silica gel: 100 to 200 mesh; eluent: acetone: petroleum ether 1:3 v:v) to give **4c** as a white solid state compound (1.31 g, yield 79%). ^1^H NMR (600 MHz, DMSO-*d*_6_, TMS), δ (ppm): 8.32 (d, *J* = 2.2 Hz, 2H), 7.65 (d, *J* = 8.8 Hz, 2H), 7.48 (dd, *J* = 8.7, 2.2 Hz, 2H), 4.38 (t, *J* = 7.0 Hz, 2H), 3.92-3.84 (m, 4H), 1.77-1.72 (m, 2H), 1.66-1.57 (m, 2H), 1.50-1.40 (m, 2H), 1.36-1.28 (m, 2H), 1.15 (t, *J* = 7.1 Hz, 6H). ^13^C NMR (150 MHz, DMSO-*d*_6_, TMS), δ (ppm): 139.0, 126.2, 123.5, 122.4, 120.4, 111.2, 60.7, 42.5, 28.1, 27.2, 27.1, 24.8, 23.9, 21.9, 16.2.

**diethyl (3-bromopropyl)phosphonate (5)** In a 100 mL dry two-necked round-bottom flask with septum, 1,3-dibromopropane (3.5 eq, 47.8 g, 24 mL, 276 mmol) was added under nitrogen protection, followed by addition of triethyl phosphite (1 eq, 13.11 g, 78.9 mmol) dropwise. A long needle was inserted through the septum and into the solution to facilitate 1-bromopropane to evolve. The reaction mixture was stirred at 160 ℃ for 2.5 hrs and then most residual 1,3-dibromopropane was removed under reduced pressure. The crude product was purified by column chromatography (silica gel: 70 to 230 mesh; eluent: first n-pentane: ethyl acetate 10:1 v:v was used to remove residual 1,3-dibromopropane, then n-pentane: ethanol from 40:1 to 30:1 v:v) to give **5** as colorless oil (3.71 g, yield 18.2%). ^1^H NMR (600 MHz, DMSO-*d*_6_, TMS), δ (ppm): 4.05-3.93 (m, 4H), 3.58 (t, *J* = 6.6 Hz, 2H), 2.03-1.95 (m, 2H), 1.89-1.81 (m, 2H), 1.23 (t, *J* = 7.0 Hz, 6H). ^13^C NMR (150 MHz, DMSO-*d*_6_, TMS), δ (ppm): 61.0, 34.9, 34.8, 26.0, 23.8, 23.0, 16.3, 16.2.

**diethyl (3-(3,6-dichloro-9*H*-carbazol-9-yl)propyl)phosphonate (6)** In a 50 mL dry two-necked round-bottom flask with mixture of 17 mL anhydrous DMSO and 3,6-dichloro-9*H*-carbazole (**2**, 1 eq, 2.0 g, 8.4 mmol), NaH (1.1 eq, 372 mg, 60% in oil, 9.3 mmol) was added. The solution become yellow color and produce some bubbles. The reaction was stirred at room temperature for 0.5 hrs, and then the solution became colorless. Diethyl (3-bromopropyl)phosphonate (**5**, 1.1 eq, 2.4 g, 9.3 mmol) was added into the solution. The reaction mixture was stirred at 60 ℃ overnight. After completion of the reaction, water was added into the mixture to quench the reaction followed by extraction with ethyl acetate. The organic layer was washed with brine for three times, dried over anhydrous Na_2_SO_4_, and then the solvent was distilled off under reduced pressure to obtain brown oil. The crude product was purified by column chromatography (silica gel: 70 to 230 mesh; eluent: ethyl acetate) to give **5** as a white solid compound (2.73 g, yield 79%). ^1^H NMR (600 MHz, DMSO-*d*_6_, TMS), δ (ppm): 8.33 (d, *J* = 2.2 Hz, 2H), 7.70 (d, *J* = 8.7 Hz, 2H), 7.50 (dd, *J* = 8.7, 2.2 Hz, 2H), 4.47 (t, *J* = 7.1 Hz, 2H), 3.98-3.88 (m, 4H), 1.96-1.88 (m, 2H), 1.78-1.68 (m, 2H), 1.16 (t, *J* = 7.1 Hz, 6H). ^13^C NMR (150 MHz, DMSO-*d*_6_, TMS), δ (ppm): 138.9, 126.3, 123.7, 122.5, 120.5, 111.1, 61.0, 42.5, 42.4, 22.3, 21.9, 21.4, 16.2.

**(2-(3,6-dichloro-9*H*-carbazol-9-yl)ethyl)phosphonic acid (2Cl-2PACz)** In a 50 mL dry round-bottom flask, (2-(3,6-dichloro-9*H*-carbazol-9-yl)ethyl)phosphonate (**4a**, 1 eq, 0.9 g, 2.3 mmol) was dissolved in anhydrous 1,4-dioxane (25 mL) under nitrogen atmosphere and bromotrimethylsilane (10 eq, 3.52 g, 3.0 mL, 23.0 mmol) was added dropwise. The reaction was stirred for 12 h at room temperature under nitrogen atmosphere. Afterwards most solvent was removed under reduced pressure, and the viscous liquid residue was dissolved in methanol (5 ml) and stirred for 0.5 hrs. Next, distilled water was added dropwise (50 ml), until solution became opaque and stirred for another 12 hrs. Product was filtered off and washed with water to give **2Cl-2PACz** as a white solid compound (720 mg, yield 93%). ^1^H NMR (600 MHz, DMSO-*d*_6_, TMS), δ (ppm): 8.33 (d, *J* = 2.5 Hz, 2H), 7.59 (d, *J* = 8.7 Hz, 2H), 7.51 (dd, *J* = 8.7, 2.2 Hz, 2H), 4.56-4.52 (m, 2H), 2.08-1.98 (m, 2H). ^13^C NMR (150 MHz, DMSO-*d*_6_, TMS), δ (ppm): 138.5, 126.4, 123.7, 122.7, 120.5, 111.0, 37.8, 27.6, 26.8.

**(3-(3,6-dichloro-9*H*-carbazol-9-yl)propyl)phosphonic acid (2Cl-3PACz)** The same prepared procedure for synthesizing **2Cl-2PACz**. The target compound **2Cl-3PACz** was finally obtained through recrystallization from ethanol as a white solid compound (0.6 g, yield 35%). ^1^H NMR (600 MHz, DMSO-d6, TMS), δ (ppm): 8.33 (d, *J* = 2.2 Hz, 2H), 7.71 (d, *J* = 8.7 Hz, 2H), 7.49 (dd, *J* = 8.7, 2.2 Hz, 2H), 4.48 (t, *J* = 7.0 Hz, 2H), 2.00-1.89 (m, 2H), 1.57-1.46 (m, 2H). ^13^C NMR (150 MHz, DMSO-*d*_6_, TMS), δ (ppm): 139.0, 126.3, 123.6, 122.4, 120.5, 111.3, 42.8, 42.7, 25.3, 24.3, 22.6.

**(4-(3,6-dichloro-9*H*-carbazol-9-yl)butyl)phosphonic acid (2Cl-4PACz)** The same prepared procedure for synthesizing **2Cl-2PACz**. The target compound **2Cl-4PACz** was finally obtained through recrystallization from ethanol as a white solid compound (0.38 g, yield 29%). ^1^H NMR (600 MHz, DMSO-d6, TMS), δ (ppm): 8.33 (d, *J* = 2.0 Hz, 2H), 7.69 (d, *J* = 8.8 Hz, 2H), 7.49 (dd, *J* = 8.7, 2.3 Hz, 2H), 4.40 (t, *J* = 7.2 Hz, 2H), 1.81 (p, *J* = 7.2 Hz, 2H), 1.56-1.44 (m, 4H). ^13^C NMR (150 MHz, DMSO-*d*_6_, TMS), δ (ppm): 139.0, 126.2, 123.5, 122.4, 120.4, 111.3, 42.3, 29.5, 29.4, 27.7, 26.8, 20.3.

**(5-(3,6-dichloro-9*H*-carbazol-9-yl)pentyl)phosphonic acid (2Cl-5PACz)** The same prepared procedure for synthesizing **2Cl-2PACz**. The target compound **2Cl-5PACz** was finally obtained as a white solid compound (0.86 g, yield 78%). ^1^H NMR (600 MHz, DMSO-*d*_6_, TMS), δ (ppm): 8.33 (d, *J* = 2.3 Hz, 2H), 7.65 (d, *J* = 8.7 Hz, 2H), 7.49 (dd, *J* = 8.7, 2.3 Hz, 2H), 4.37 (t, *J* = 7.3 Hz, 2H), 1.73 (p, *J* = 7.4 Hz, 2H), 1.51-1.39 (m, 4H), 1.34 (p, *J* = 7.5 Hz, 2H). ^13^C NMR (150 MHz, DMSO-*d*_6_, TMS), δ (ppm): 138.9, 126.2, 123.5, 122.4, 120.5, 111.2, 42.5, 28.1, 27.7, 27.4, 27.4, 27.1, 22.5.

**3. Supporting Figures S1 to S22 & Tables S1 to S11**

**Figure S1.** Thermogravimetric analysis (TGA) spectra of 2Cl-2PACz, 2Cl-3PACz, 2Cl-4PACz and 2Cl-5PACz; heating rate: 10 ℃ min^-1^, from 30 ℃ to 500 ℃ under nitrogen atmosphere.

**Table S1.** TGA data of 2Cl-2PACz, 2Cl-3PACz, 2Cl-4PACz and 2Cl-5PACz for 5% weight loss.

|  | 2Cl-2PACz | 2Cl-3PACz | 2Cl-4PACz | 2Cl-5PACz |
| --- | --- | --- | --- | --- |
| T (ºC) | 337 | 339 | 338 | 366 |

**Figure S2.** (**A**) Schematic process for SAMs adsorption on quartz glass and UV-vis measurements. **I.** Fabrication of 2Cl-2PACz-spin-coated quartz glass (SAMs in MeOH of 0.3 mg mL^-1^). **II.** Thermal annealing of SAMs-modified ITO glass. **III.** UV-vis spectroscopy measurement of 2Cl-2PACz-spin-coated quartz glass. **IV.** Rinsing quartz/2Cl-2PACz layer with methanol solution. **V.** UV-vis spectroscopy measurement of methanol rinsed quartz glass. (**B**) and (**D**) UV-vis spectra of blank quartz (black dash line) and spin-coated quartz substrates at varying concentrations of 2Cl-2PACz (0.1, 0.2, 0.3, 0.4, 0.6, and 0.8 mg mL⁻¹ in MeOH), both before (thick solid line) and after rinsing with methanol solution (thin solid line). (**C**) and (**E**) Estimated number of 2Cl-2PACz layers on spin-coated quartz glass at varying concentrations without rinsing.

Quartz glass was used instead of ITO glass here due to its lower absorbance in the UV region. Quartz glass was pre-cleaned and subjected to plasma treatment in the same manner as ITO glass, resulting in similar hydroxyl groups on the surface to facilitate interaction with the SAMs. Firstly, it was observed that as the concentration of the 2Cl-2PACz solution increases, the absorbance intensity across the entire spectrum generally rises, indicating the formation of multi-layer structures on quartz. Futhermore, after rinsing with methanol to remove the possible residual SAMs on the first monolayer, an evidently decreased absorbance intensity for all the films were observed. This suggests that mutlti-layers of 2Cl-2PACz can be removed by methanol washing. Additionally, we observed that after two or more times of washing with methanol, the absorbance intensity at 304 nm of quartz/2Cl-2PACz (0.3 mg mL^-1^) almost doesn’t change anymore, indicating the formation of monolayer. This is due to the strong covalent bonds between the phosphate groups of the SAMs and the hydroxyl groups on the quartz glass. The net absorbance value (subtracting the quartz background) of monolayer was found in the range of 0.023 to 0.027 at 304 nm for repeated experiments.

Based on the above observation, we can estimate the number of layers of quartz/2Cl-2PACz prepared with different concentrations by analyzing the net absorbance intensity at 304 nm. Other peaks at 239 and 270 nm are not considered due to the relatively high background absorption of quartz. It was found that the quartz/2Cl-2PACz films contained approximately 1-3 layers, 2-4 layers, and 3-5 layers for samples prepared from concentrations of 0.1, 0.2, and 0.3 mg mL⁻¹, respectively. However, when the SAMs concentration exceeded 0.4 mg mL⁻¹, a significantly elevated baseline from 400 to 800 nm and a pronounced absorption peak at 304 nm were observed. This indicates that the film is relatively thick, which cause strong reflections.

**Figure S3.** UV-vis spectra of blank quartz (black dash line) and spin-coated quartz substrates with **4a** (0.3 mg mL⁻¹ in MeOH), both before and after rinsing with methanol solution.

**Density functional theory (DFT) calculation:**

**Molecular calculations**

The electronic structures of 2Cl-2PACz, 2Cl-3PACz, 2Cl-4PACz and 2Cl-5PACz molecules were systematically investigated using DFT and Time-Dependent DFT (TD-DFT) methods. Geometry optimizations and all the electronic properties calculations were performed at the B3LYP^[4]^/6-311G(d,p)^[5]^ theory level. For simplicity, the phosphonic acid (-PO(OH)_2_) binding group was substituted by a hydrogen atom. The energy levels of the systems were determined by calculating the energy of the frontier molecular orbitals and the energy of the first electronic transition (S1). The molecular calculations were carried out using the Gaussian 16 program (Rev C.01).^[6]^

**Adsorption of SAMs on ITO surface**

Density functional theory calculations were performed to obtain the work function of ITO with and without absorbed 2Cl-2PACz, 2Cl-3PACz, 2Cl-4PACz and 2Cl-5PACz molecules. The ITO was modeled using a slab with In_60_Sn_4_O_96_ stoichiometry, featuring approximately 27 Å of vacuum along the z-axis. The molecules were adsorbed on only one side of the slab. To solve the Kohn-Sham equations, we utilized the Perdew-Burke-Ernzerhof (PBE) generalized gradient approximation^[7]^ in conjunction with the all-electron projector augmented-wave (PAW) method.^[8]^ The equilibrium volume of the In_60_Sn_4_O_96_ slabs with and without adsorbed molecules was found by optimizing the stress tensor and atomic forces within a plane-wave cutoff energy of 500 eV along the integration over the Brillouin zone using a k-mesh of 5x5x1. The equilibrium crystal structures were reached once the atomic forces on every atom were smaller than 0.010 eV Å^-1^ and using self-consistent criteria of 10^-5^ eV for the total energy. To obtain the work function, the precision of the calculations was increased by using a k-mesh of 7x7x1. The calculations were carried out using the Vienna ab initio simulation package (VASP),^[9]^ 11 version 5.4.1.

**
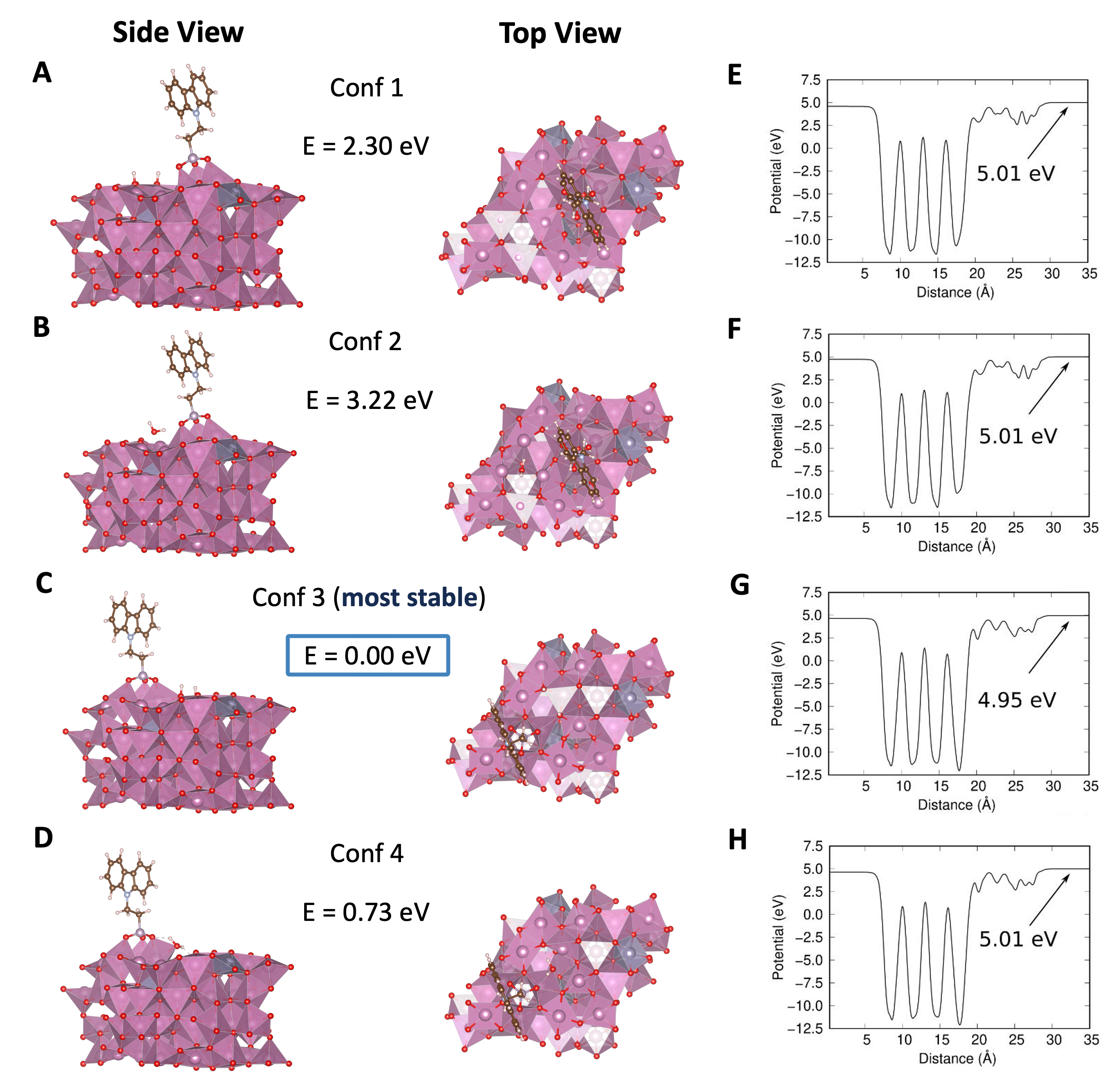
**

**Figure S4.** (**A**)-(**D**) Side and top views of different adsorption configurations for SAMs of 2PACz on ITO (111) surface, the values for “E” give the energy of each system referenced to the energy of the most stable one (**C**). (**E**)-(**H**) The electrostatic potential energy profile for each system, with the Fermi energy level shifted to zero and numbers showing the shifted work functions of ITO by the adsorbed molecules. Conformations 1 and 3 present two hydrogens absorbed on surface and conformations 2 and 4 present a water molecule adsorbed on surface. Unlike conformations 1 and 2, conformations 3 and 4 do not have an oxygen atom just below the phosphate group.

Through DFT simulations, we found that conformation 3 offered the lowest energy for the system. Consequently, all subsequent DFT calculations on work functions were based on this conformation.

**Figure S5.** (**A**)-(**D**) View of the most stable adsorption configurations for SAMs of 2Cl-2PACz, 2Cl-3PACz, 2Cl-4PACz and 2Cl-5PACz on ITO (111) surface. (**E**)-(**H**) The electrostatic potential energy profile for each system, with the Fermi energy level shifted to zero and blue numbers showing the shifted work functions of ITO by the adsorbed molecules. The simulated angles between carbazole dipole moment and its projection on ITO surface in the optimized geometries (without temperature effects) were 69.7° for 2Cl-2PACz, 22.9° for 2Cl-3PACz, 75.4° for 2Cl-4PACz, 28.6° for 2Cl-5PACz.

**Figure S6.** (**A**) Calculated shifted work function of ITO by SAMs of 2Cl-2PACz, 2Cl-3PACz, 2Cl-4PACz and 2Cl-5PACz in the most stable conformation, and experimental values presented for comparison. (**B**) Calculated work functions versus the distance of the closest chloride atom to ITO surface. (**C**) Calculated work functions versus the dipole moment component perpendicular to the ITO surface.


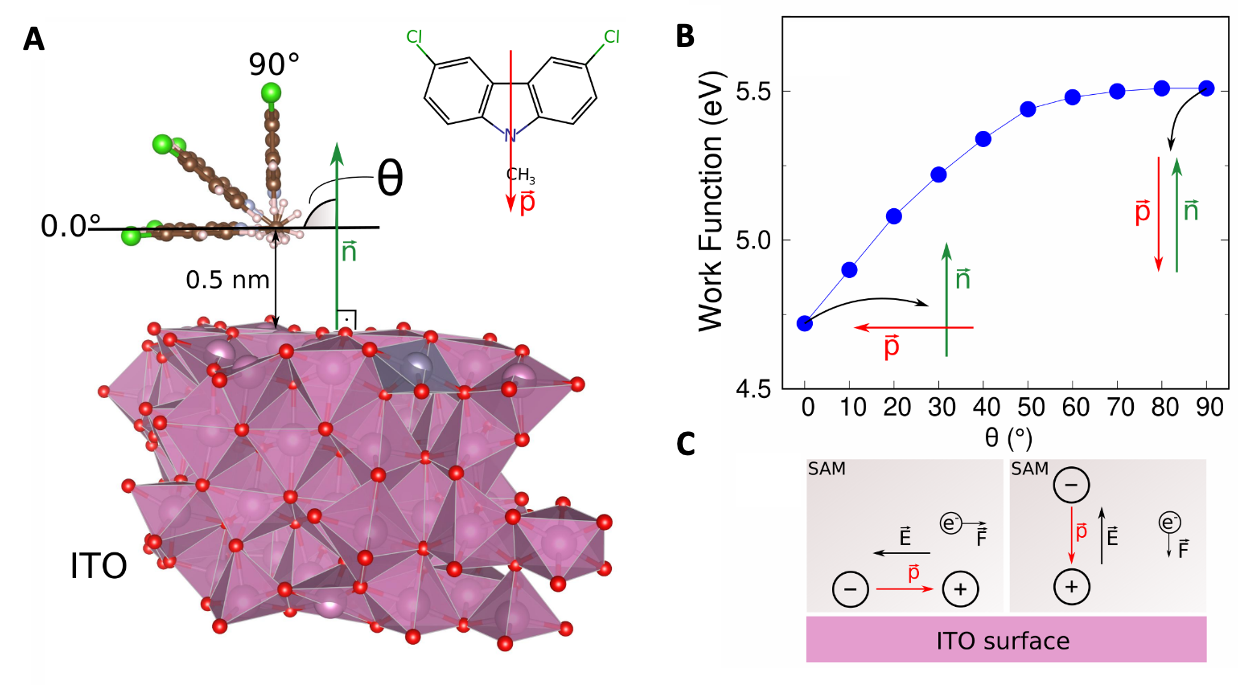


**Figure S7.** (**A**) and (**B**) Angular dependence of calculated work function with a dichloride-substituted carbazole group dipole moment. The work function exhibits variation relative to the angle between the carbazole dipole moment vector (**p**) and the vector perpendicular to the ITO surface (**n**). Maximum work function occurs when **p** aligns antiparallel to **n**, while minimum work function occurs when **p** is perpendicular to **n**. (**C**) Illustration of the electric field (**E**) vector and the electrical force acting in a negative particle in the surface of ITO given to the presence of the molecular dipole moment (**p**).

**Figure S8.** Conformational dependence of calculated WFs for 2Cl-3PACz on ITO surfaces. (**A**)-(**F**) Optimized geometries depict various conformations of the system. In conformations (**A**)-(**C**) and (**D**)-(**F**) the 2Cl-3PACz molecule is placed in two different regions over ITO surface. The values for “E” give the energy of each system referenced to the energy of the most stable one. In conformations **A**, **B**, **D**, and **E**, the carbazole plane is nearly parallel to the ITO surface, whereas in conformations **C** and **F**, it exhibits a more inclined orientation.

**Table S2.** Summary of optical parameters of 2Cl-2PACz, 2Cl-3PACz, 2Cl-4PACz and 2Cl-5PACz in ethanol solution from **Figure 2D**.

|  | 2Cl-2PACz | 2Cl-3PACz | 2Cl-4PACz | 2Cl-5PACz |
| --- | --- | --- | --- | --- |
| λ_max_ (nm) | 360 | 361 | 363 | 363 |
| ɛ (M^-1^ cm^-1^) | 3.62×10^3^ | 3.67×10^3^ | 3.61×10^3^ | 3.63×10^3^ |
| λ_onset_ (nm) | 372 | 372 | 374 | 374 |
| E_g, opt._ (eV) | 3.33 | 3.33 | 3.32 | 3.32 |

**Figure S9.** Hole and electron DFT density plots (isovalue = 0.05) of the first excited state (S1) and the most intense one (S_λ_^max^) of 2Cl-2PACz, 2Cl-3PACz, 2Cl-4PACz and 2Cl-5PACz in ethanol.


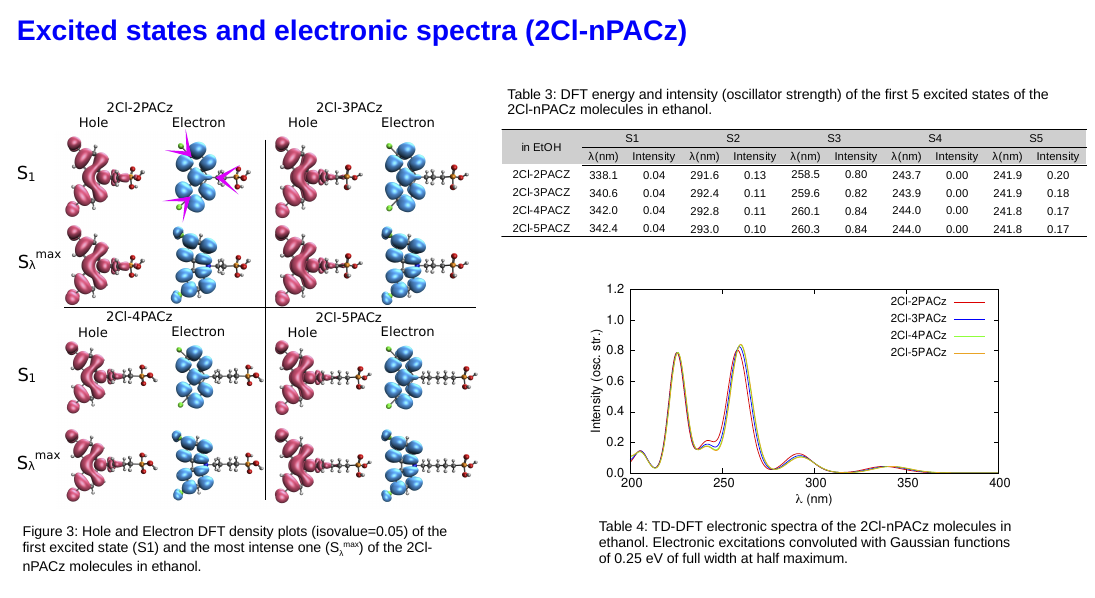


**Figure S10.** TD-DFT electronic spectra of 2Cl-2PACz, 2Cl-3PACz, 2Cl-4PACz and 2Cl-5PACz in ethanol. Electronic excitions convoluted with Gaussian functions of 0.25 eV of full width at half maximum.

**Table S3.** DFT energy and intensity (oscillator strength) of the first 5 excited states (S1 to S5) of 2Cl-2PACz, 2Cl-3PACz, 2Cl-4PACz and 2Cl-5PACz in ethanol, and compared with maximum absorption wavelength in measured UV-vis absorption spectra.

| SAMs | Exp. | S1 | | S2 | | S3 | | S4 | | S5 | |
| --- | --- | --- | --- | --- | --- | --- | --- | --- | --- | --- | --- |
|  | 𝜆 (nm) | 𝜆 (nm) | Intensity | 𝜆 (nm) | Intensity | 𝜆 (nm) | Intensity | 𝜆 (nm) | Intensity | 𝜆 (nm) | Intensity |
| 2Cl-2PACz | 360 | 338.1 | 0.04 | 291.6 | 0.13 | 258.5 | 0.80 | 243.7 | 0.00 | 241.9 | 0.20 |
| 2Cl-3PACz | 361 | 340.6 | 0.04 | 292.4 | 0.11 | 259.6 | 0.82 | 243.9 | 0.00 | 241.9 | 0.18 |
| 2Cl-4PACz | 363 | 342.0 | 0.04 | 292.8 | 0.11 | 260.1 | 0.84 | 244.0 | 0.00 | 241.8 | 0.17 |
| 2Cl-5PACz | 363 | 342.4 | 0.04 | 293.0 | 0.10 | 260.3 | 0.84 | 244.0 | 0.00 | 241.8 | 0.17 |


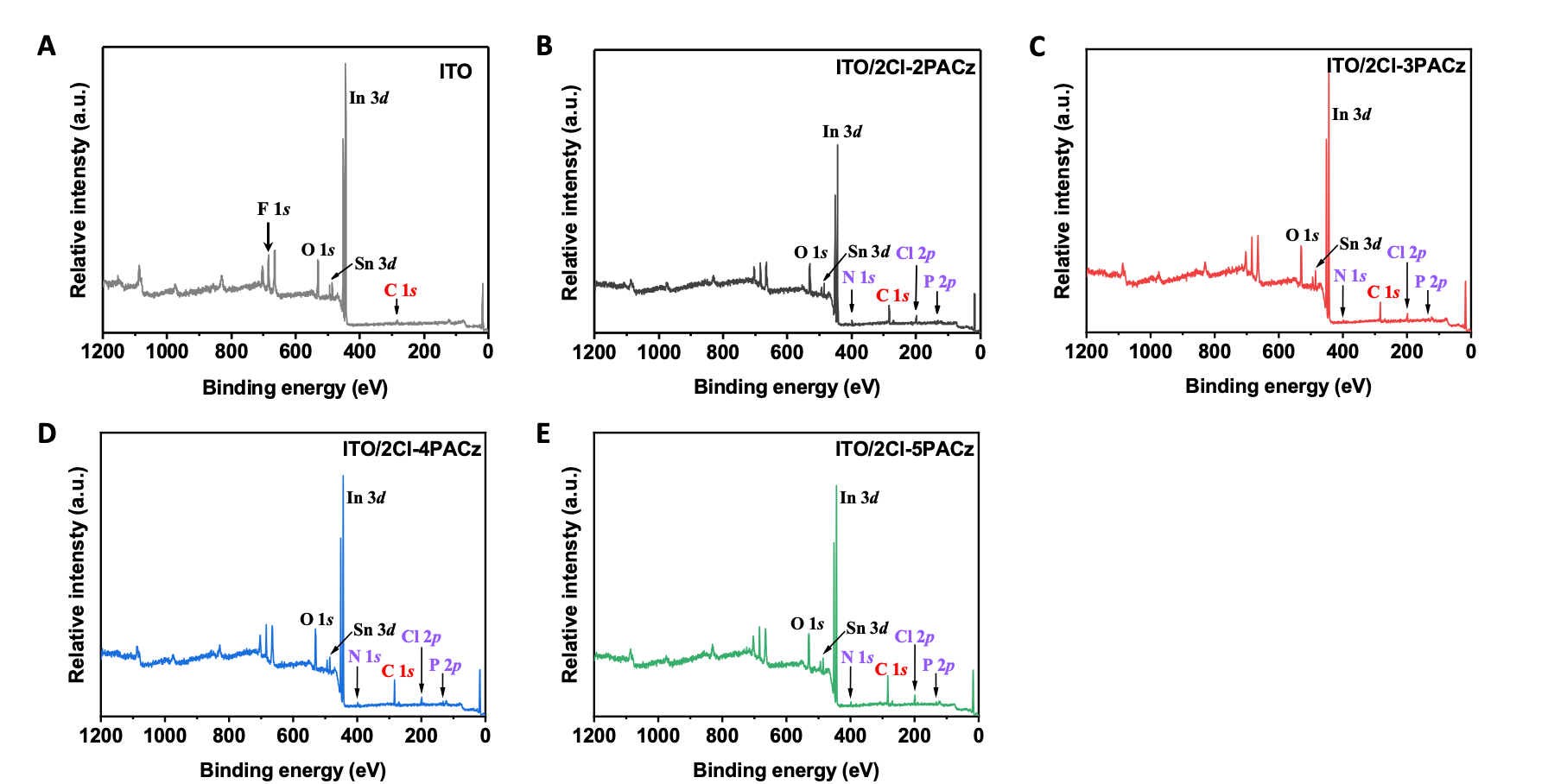


**Figure S11.** HR-XPS surveys of (**A**) bare ITO, (**B**) 2Cl-2PACz, (**C**) 2Cl-3PACz, (**D**) 2Cl-4PACz and (**E**) 2Cl-5PACz-modified ITO.

**Figure S12.** HR-XPS spectroscopy of C 1*s* for different SAMs, (**A**) 2Cl-2PACz, (**B**) 2Cl-3PACz, (**C**) 2Cl-4PACz and (**D**) 2Cl-5PACz-modified ITO.

**Table S4.** C 1*s*, Cl 2*p*, P 2*p*, N 1*s* and In 3*d*_3/2_ core-level peak area as measured by HR-XPS for different SAMs-modified ITO. The C 1*s* peak area and Cl 2*p* peak area are divided by the number of carbon atoms and chloride atoms, and a relative coverage factor is calculated by normalizing to the In 3*d*_3/2_ core-level area.

| SAMs | XPS Peak Area | | | | | # atoms | Coverage Factor | | | |
| --- | --- | --- | --- | --- | --- | --- | --- | --- | --- | --- |
|  | C 1*s* | Cl 2*p* | N 1*s* | P 2*p* | In 3*d_3/2_* | C/Cl | C 1*s*/  In 3*d_3/2_* | Cl 2*p*/  In 3*d_3/2_* | N 1*s*/  In 3*d_3/2_* | P 2*p*/  In 3*d_3/2_* |
| 2Cl-2PACz | 1215.5 | 279.2 | 159.5 | 158.3 | 7182.3 | 14/2 | 1.2∙10^-2^ | 1.9∙10^-2^ | 2.2∙10^-2^ | 2.2∙10^-2^ |
| 2Cl-3PACz | 1198.9 | 245.8 | 147.0 | 153.7 | 10450.5 | 15/2 | 7.7∙10^-3^ | 1.2∙10^-2^ | 1.4∙10^-2^ | 1.5∙10^-2^ |
| 2Cl-4PACz | 1516.2 | 283.4 | 167.4 | 162.1 | 9439.3 | 16/2 | 1.0∙10^-2^ | 1.5∙10^-2^ | 1.8∙10^-2^ | 1.7∙10^-2^ |
| 2Cl-5PACz | 1632.1 | 301.1 | 173.9 | 165.0 | 9153.3 | 17/2 | 1.1∙10^-2^ | 1.6∙10^-2^ | 1.9∙10^-2^ | 1.8∙10^-2^ |

**Figure S13.** UV-vis absorption spectra of blank quartz glass and SAMs-spin coated quartz glass before and after washing twice with methanol solution.


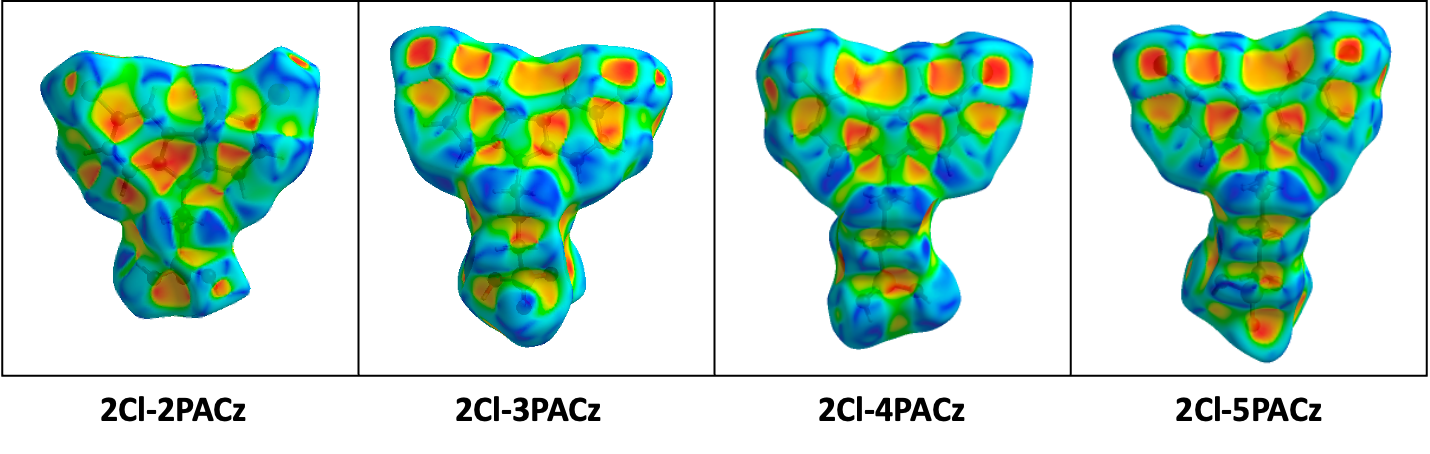


**Figure S14.** Shape index/Hirshfeld surface analysis diagram of 2Cl-2PACz, 2Cl-3PACz, 2Cl-4PACz and 2Cl-5PACz.

**Table S5.** Summary of intermolecular interaction percentage between atoms from Hirshfeld surface and fingerprint plots analysis from **Figure S15**.

| SAMs | Intermolecular interaction percentage | | | | | | | | | | |
| --- | --- | --- | --- | --- | --- | --- | --- | --- | --- | --- | --- |
|  | C···H | H···C | H···H | C···C | C···Cl | Cl···C | Cl···H | H···Cl | O···H | H···O | Cl···Cl |
| 2Cl-2PACz | 13.5% | 9.9% | 24.3% | 1.2% | 3.2% | 2.7% | 12.3% | 7.5% | 10.2% | 7.1% | 2.7% |
| 2Cl-3PACz | 10.1% | 7.7% | 21.9% | 3.3% | 3.0% | 2.9% | 15.3% | 11.5% | 12.2% | 10.6% | 0.0% |
| 2Cl-4PACz | 8.7% | 6.6% | 29.9% | 3.9% | 2.4% | 2.4% | 14.8% | 11.1% | 9.7% | 8.5% | 0.0% |
| 2Cl-5PACz | 8.4% | 6.7% | 32.2% | 3.3% | 2.5% | 2.4% | 14.2% | 10.3% | 10.1% | 8.4% | 0.0% |

**Figure S15.** Interaction fingerprint of representative atom interactions for 2Cl-2PACz, 2Cl-3PACz, 2Cl-4PACz and 2Cl-5PACz, *di* and *de* denote the closest distances from a point on the Hirshfeld surface to atoms inside and outside the surface, respectively.


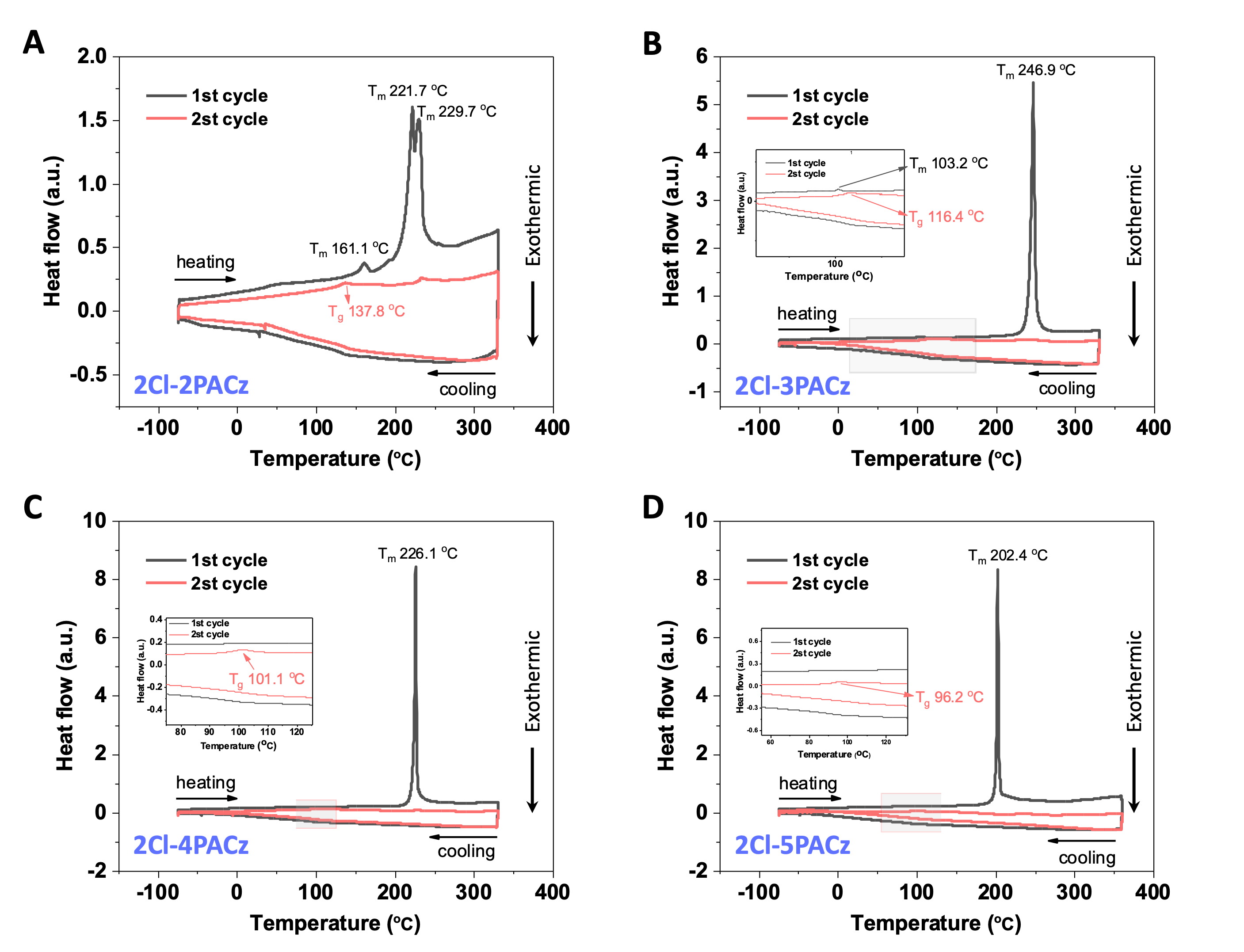


**Figure S16.** Differential scanning calorimetry (DSC) spectra of SAMs, (**A**) 2Cl-2PACz, (**B**) 2Cl-3PACz, (**C**) 2Cl-4PACz and (**D**) 2Cl-5PACz for 2 cycles. Heating/cooling rate: 10 ℃ min^-1^, under nitrogen atmosphere. (T_m_: temperature of melting point; T_g_: temperature of glass transition point)

**Figure S17.** Distances (unit: Å) between chlorine atoms and the plane of the three oxygen atoms in phosphate group of 2Cl-2PACz, 2Cl-3PACz, 2Cl-4PACz and 2Cl-5PACz from single crystals.

**Figure S18.** AFM hight images of 2Cl-2PACz, 2Cl-3PACz, 2Cl-4PACz and 2Cl-5PACz on ITO surfaces.

**
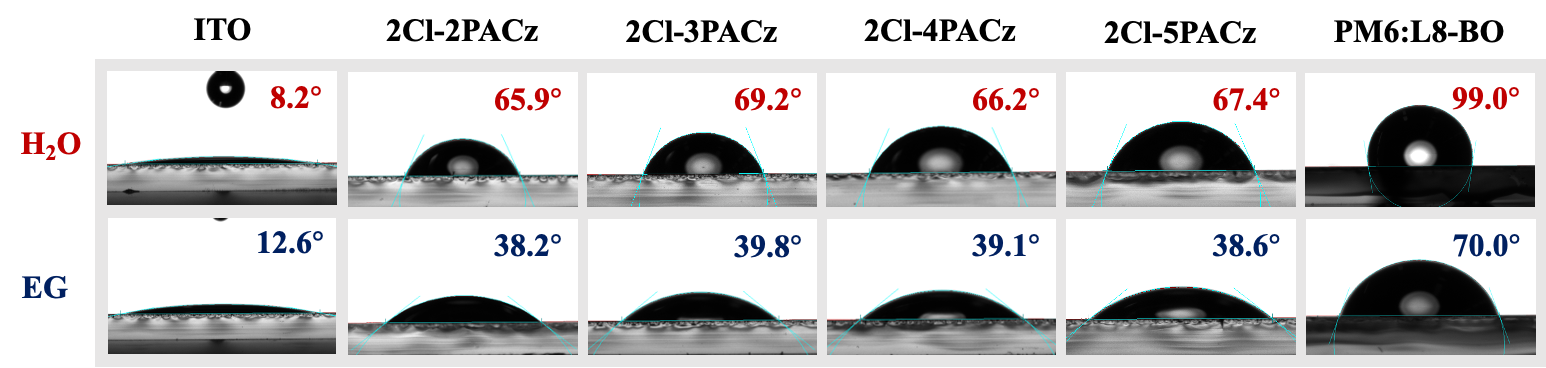
**

**Figure S19.** Contact angle images from bare ITO, ITO glass with diverse SAMs and PM6:L8-BO. (EG: ethylene glycol)

**Table S6.** Polarity and dispersion force parameters of H_2_O and EG.

| Liquid | $\gamma_{l}^{p}$ | $\gamma_{l}^{D}$ | $\gamma_{l}$ | ${\gamma_{l}^{P}}/{\gamma_{l}^{D}}$ |
| --- | --- | --- | --- | --- |
| H_2_O | 51 | 21.8 | 72.8 | 2.36 |
| EG | 19 | 29.0 | 48.0 | 0.66 |

**Table S7.** Parameters of contact angle and surface energy bare ITO, ITO glass with diverse SAMs and PM6:L8-BO.

| ITO glass modified with SAMs or PM6:L8-BO | Contact angle ($^{\circ}$) | | Surface energy  $\gamma$ (mJ m^-2^) |
| --- | --- | --- | --- |
|  | H_2_O | EG |  |
| bare ITO | 8.2 | 12.6 | 88.33 |
| 2Cl-2PACz | 65.9 | 38.2 | 38.42 |
| 2Cl-3PACz | 69.2 | 39.8 | 37.55 |
| 2Cl-4PACz | 66.2 | 39.1 | 38.03 |
| 2Cl-5PACz | 67.4 | 38.6 | 38.09 |
| PM6:L8-BO | 99.0 | 70.0 | 28.90 |

**Figure S20.** The equivalent-circuit model employed for EIS fitting of the devices. *R*_ele_ corresponds to electrode resistance including ITO and Ag; *R*_int_ and *C*_int_ parallel connection corresponds to resistance and capacitance of the interface layer; *R*_bhj_ and *C*_bhj_ parallel connection corresponds to resistance and capacitance of the BHJ layer.

**Table S8.** The fitting parameters of OSCs from Nyquist plots.

| HTLs | *R*_ele, int, bhj_ [Ω] | *R*_ele_ [Ω] | *R*_int_ [Ω] | *C*_int_ [nF] | *R*_bhj_ [Ω] | *C*_bhj_ [nF] |
| --- | --- | --- | --- | --- | --- | --- |
| 2Cl-2PACz | 260.4 | 44.7 | 100.7 | 4.5 | 114.1 | 26.9 |
| 2Cl-3PACz | 475.4 | 53.2 | 182.6 | 3.9 | 237.1 | 27.5 |
| 2Cl-4PACz | 1585.4 | 66.7 | 845.7 | 3.4 | 676.3 | 17.7 |
| 2Cl-5PACz | 2106.4 | 57.7 | 995.7 | 2.8 | 1045.0 | 24.3 |

**Figure S21.** Mott Schottky curves of OSC devices based on 2Cl-2PACz, 2Cl-3PACz, 2Cl-4PACz and 2Cl-5PACz.

**Figure S22.** *J*-*V* characteristics of the OSCs with different HTLs in (**A**) PM6:BTP-eC9 and (**B**) PM6:Y6 BHJ systems.

**Table S9.** Photovoltaic parameters of OSCs based on PM6:BTP-eC9 and PM6:Y6 BHJs with different SAMs HTLs measured under illumination with AM 1.5G (100 mW cm^-2^).

| Active layer | HTLs | *V*_oc_  [V] | *J*_sc_  [mA cm^-2^] | FF  [%] | PCE ^a)^  [%] | *R*_s_  [Ω] |
| --- | --- | --- | --- | --- | --- | --- |
| PM6:BTP-eC9 | 2Cl-2PACz | 0.859 | 28.31 | 74.25 | 18.06 (17.85 ± 0.14) | 40.8 |
|  | 2Cl-3PACz | 0.851 | 28.34 | 64.82 | 15.43 (14.81 ± 0.46) | 93.5 |
|  | 2Cl-4PACz | 0.847 | 28.78 | 62.39 | 15.20 (14.47 ± 0.61) | 95.44 |
|  | 2Cl-5PACz | 0.838 | 28.16 | 53.57 | 12.64 (11.79 ± 0.56) | 140.05 |
| PM6:Y6 | 2Cl-2PACz | 0.844 | 27.89 | 72.02 | 16.95 (16.75 ± 0.18) | 37.6 |
|  | 2Cl-3PACz | 0.834 | 27.77 | 60.68 | 14.05 (13.80 ± 0.27) | 102.8 |
|  | 2Cl-4PACz | 0.826 | 28.07 | 59.30 | 13.75 (13.44 ± 0.27) | 94.4 |
|  | 2Cl-5PACz | 0.817 | 28.49 | 50.49 | 11.76 (11.27 ± 0.36) | 158.4 |

^a)^ The average PCE values in brackets were obtained from 5 devices.

**Table S10.** Photovoltaic parameters of OSCs based on PM6:L8-BO with different treatment of SAMs HTLs measured under illumination with AM 1.5G (100 mW cm^-2^).

| HTLs | *V*_oc_  [V] | *J*_sc_  [mA cm^-2^] | FF  [%] | PCE ^a)^  [%] | *R*_s_  [Ω] |
| --- | --- | --- | --- | --- | --- |
| 2Cl-2PACz | 0.880 | 27.32 | 77.44 | 18.62 (17.94 ± 0.39) | 31.1 |
| 2Cl-2PACz/MeOH twice | 0.894 | 27.59 | 76.79 | 18.95 (18.46 ± 0.51) | 62.6 |
| 2Cl-2PACz/SAMs once | 0.896 | 24.38 | 71.14 | 15.53 (14.52 ± 0.58) | 55.7 |
| 2Cl-2PACz/ SAMs twice | 0.898 | 25.82 | 73.45 | 17.03 (14.48 ± 1.26) | 52.7 |
| 2Cl-3PACz | 0.873 | 27.03 | 71.10 | 16.61 (16.43 ± 0.25) | 93.0 |
| 2Cl-3PACz/MeOH twice | 0.891 | 27.72 | 72.21 | 17.85 (16.99 ± 0.94) | 98.8 |
| 2Cl-3PACz/ SAMs once | 0.890 | 25.72 | 67.82 | 15.52 (14.79 ± 0.51) | 99.3 |
| 2Cl-3PACz/ SAMs twice | 0.894 | 26.29 | 71.98 | 16.91 (14.90 ± 1.32) | 72.4 |

^a)^ The average PCE values in brackets were obtained from 8-10 devices.

**Table S11.** Experimental details from single crystals.

| **SAMs** | **2Cl-2PACz** | **2Cl-3PACz** | **2Cl-4PACz** | **2Cl-5PACz** |
| --- | --- | --- | --- | --- |
| CCDC No. | 2352347 | 2366230 | 2366232 | 2366233 |
| File name | (2cl_2pacz_auto) | (2cl3pacz100k_auto) | (2cl_4pacz140k_auto) | (2cl_5pcaz140k_auto) |
| **Crystal data** | | | | |
| Chemical formula | 0.229(C_14_H_12_Cl_2_NO_3_P)·0.229(H_2_O) | 2(C_15_H_14_Cl_2_NO_3_P) | C_16_H_16_Cl_2_NO_3_P | C_17_H_18_Cl_2_NO_3_P |
| *M*_r_ | 82.77 | 716.28 | 372.17 | 386.19 |
| Crystal system, space group | Orthorhombic,  *Pna*2_1_ | Orthorhombic,  *Pna*2_1_ | Monoclinic,  *P*2_1_/*n* | Monoclinic,  *P*2_1_/*c* |
| Temperature (K) | 129 | 111 | 145 | 145 |
| *a*, *b*, *c* (Å) | 17.8159 (3),  6.0586 (1),  28.6846 (4) | 18.1005 (3),  4.47058 (7),  37.6098 (7) | 9.4947 (3),  4.4415 (1),  38.6591 (8) | 20.6404 (5),  4.4802 (1),  19.1919 (4) |
| α, β, γ (°) | 90, 90, 90 | 90, 90, 90 | 90, 91.855(2),  90 | 90, 96.527 (2),  90 |
| *V* (Å^3^) | 3096.20 (8) | 3043.38 (9) | 1629.43 (7) | 1763.23 (7) |
| *Z* | 4 | 4 | 4 | 4 |
| Radiation type | Cu *K*α | Cu *K*α | Cu *K*α | Cu *K*α |
| µ (mm^−1^) | 4.91 | 4.94 | 4.66 | 4.31 |
| Crystal size (mm) | 0.13 × 0.02 × 0.01 | 0.42 × 0.06 × 0.04 | 0.47 × 0.07 × 0.02 | 0.28 × 0.04 × 0.04 |
|  | | | | |
| Data collection | | | | |
| Diffractometer | XtaLAB  Synergy R,  HyPix | XtaLAB Synergy R, HyPix | XtaLAB Synergy R, HyPix | XtaLAB  Synergy R,  HyPix |
|  |  |  |  |  |
| *T*_min_, *T*_max_ | 0.900, 1.000 | 0.469, 1.000 | 0.297, 1.000 | 0.845, 1.000 |
| No. of measured, independent and observed [*I* > 2σ(*I*)] reflections | 14451, 5356, 4684 | 15123, 5402, 5033 | 15583, 3245, 2802 | 17780, 3563, 2809 |
| *R*_int_ | 0.045 | 0.041 | 0.050 | 0.043 |
| (sin θ/λ)_max_ (Å^−1^) | 0.628 | 0.628 | 0.627 | 0.628 |
| **Refinement** | | | | |
| *R*[*F*^2^ >2σ(*F*^2^)], *wR*(*F*^2^), *S* | 0.050, 0.139, 1.06 | 0.079, 0.199, 1.11 | 0.048, 0.119, 1.14 | 0.044, 0.122, 1.03 |
| No. of reflections | 5356 | 5402 | 3245 | 3563 |
| No. of parameters | 414 | 402 | 216 | 225 |
| No. of restraints | 1 | 1 | 0 | 0 |
| H-atom treatment | H atoms treated by a mixture of independent and constrained refinement | H-atom parameters constrained | H atoms treated by a mixture of independent and constrained refinement | H atoms treated by a mixture of independent and constrained refinement |
| Δρ_max_,  Δρ_min_ (e Å^−3^) | 0.63, −0.36 | 2.88, −0.67 | 0.52, −0.44 | 0.22, −0.53 |
| Absolute structure | Refined as an inversion twin. | Refined as an inversion twin. | – | – |
| Absolute structure parameter | 0.43 (3) | 0.38 (3) | – | – |

**4. Nuclear magnetic resonance spectra:**

**Figure S23.** ^1^H NMR spectrum (600 MHz) for **2** taken in DMSO-*d*_6_ at 25 ℃.

**Figure S24.** ^13^C NMR spectrum (150 MHz) for **2** taken in DMSO-*d*_6_ at 25 ℃.

**Figure S25.** ^1^H NMR spectrum (600 MHz) for **3a** taken in DMSO-*d*_6_ at 25 ℃.

**Figure S26.** ^13^C NMR spectrum (150 MHz) for **3a** taken in DMSO-*d*_6_ at 25 ℃.

**Figure S27.** ^1^H NMR spectrum (600 MHz) for **3b** taken in DMSO-*d*_6_ at 25 ℃.

**Figure S28.** ^13^C NMR spectrum (150 MHz) for **3b** taken in DMSO-*d*_6_ at 25 ℃.

**Figure S29.** ^1^H NMR spectrum (600 MHz) for **3c** taken in DMSO-*d*_6_ at 25 ℃.

**Figure S30.** ^13^C NMR spectrum (150 MHz) for **3c** taken in DMSO-*d*_6_ at 25 ℃.

**Figure S31.** ^1^H NMR spectrum (600 MHz) for **4a** taken in DMSO-*d*_6_ at 25 ℃.

**Figure S32.** ^13^C NMR spectrum (150 MHz) for **4a** taken in DMSO-*d*_6_ at 25 ℃.

**Figure S33.** ^1^H NMR spectrum (600 MHz) for **4b** taken in DMSO-*d*_6_ at 25 ℃.


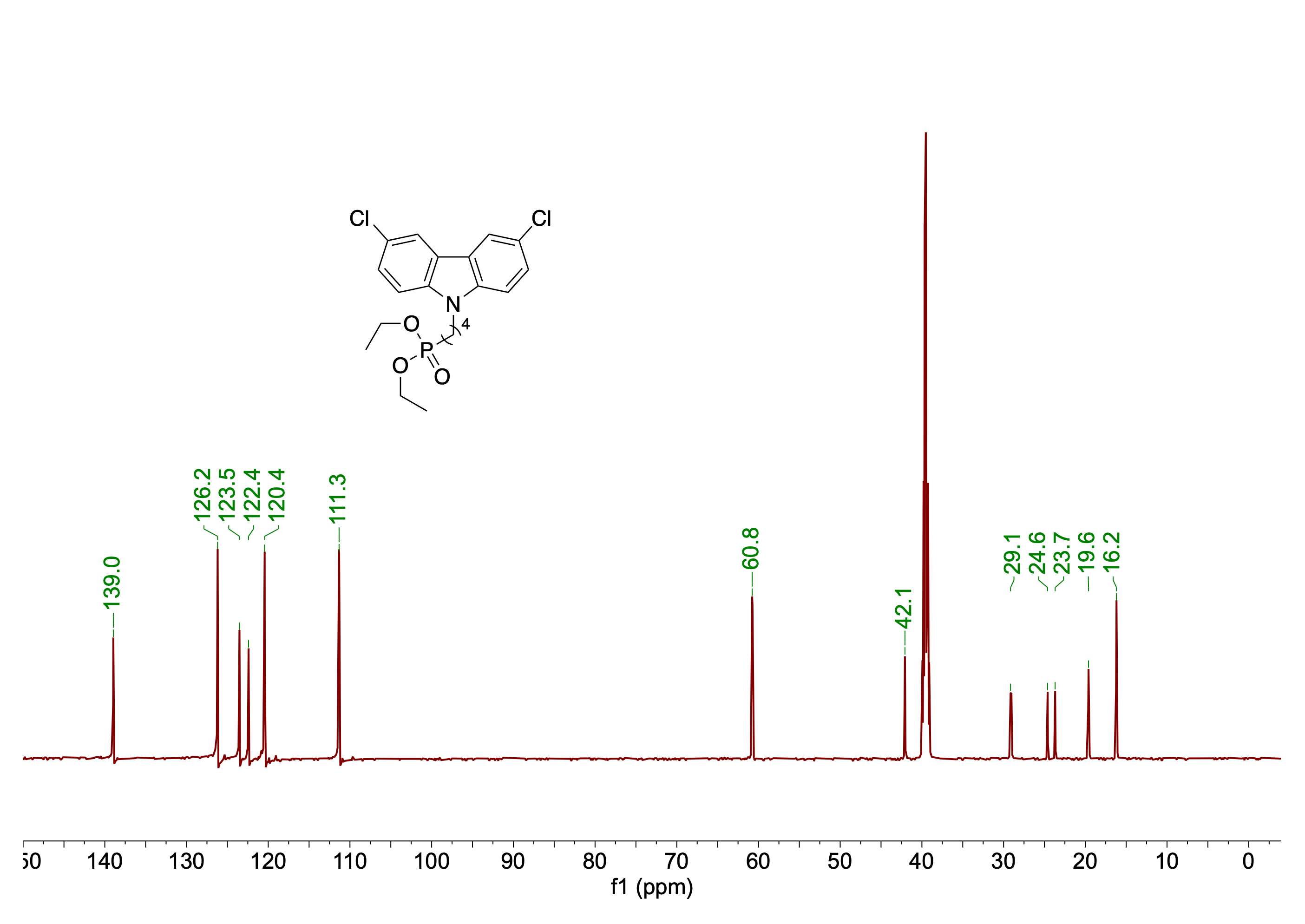


**Figure S34.** ^13^C NMR spectrum (150 MHz) for **4b** taken in DMSO-*d*_6_ at 25 ℃.

**Figure S35.** ^1^H NMR spectrum (600 MHz) for **4c** taken in DMSO-*d*_6_ at 25 ℃.

**Figure S36.** ^13^C NMR spectrum (150 MHz) for **4c** taken in DMSO-*d*_6_ at 25 ℃.

**Figure S37.** ^1^H NMR spectrum (600 MHz) for **5** taken in DMSO-*d*_6_ at 25 ℃.

**Figure S38.** ^13^C NMR spectrum (150 MHz) for **5** taken in DMSO-*d*_6_ at 25 ℃.

**Figure S39.** ^1^H NMR spectrum (600 MHz) for **6** taken in DMSO-*d*_6_ at 25 ℃.

**Figure S40.** ^13^C NMR spectrum (150 MHz) for **6** taken in DMSO-*d*_6_ at 25 ℃.

**Figure S41.** ^1^H NMR spectrum (600 MHz) for **2Cl-2PACz** taken in DMSO-*d*_6_ at 25 ℃.

**Figure S42.** ^13^C NMR spectrum (150 MHz) for **2Cl-2PACz** taken in DMSO-*d*_6_ at 25 ℃.

**Figure S43.** ^1^H NMR spectrum (600 MHz) for **2Cl-3PACz** taken in DMSO-*d*_6_ at 25 ℃.

**Figure S44.** ^13^C NMR spectrum (150 MHz) for **2Cl-3PACz** taken in DMSO-*d*_6_ at 25 ℃.

**Figure S45.** ^1^H NMR spectrum (600 MHz) for **2Cl-4PACz** taken in DMSO-*d*_6_ at 25 ℃.

**Figure S46.** ^13^C NMR spectrum (150 MHz) for **2Cl-4PACz** taken in DMSO-*d*_6_ at 25 ℃.

**Figure S47.** ^1^H NMR spectrum (600 MHz) for **2Cl-5PACz** taken in DMSO-*d*_6_ at 25 ℃.

**Figure S48.** ^13^C NMR spectrum (150 MHz) for **2Cl-5PACz** taken in DMSO-*d*_6_ at 25 ℃.

**5. Supplemental references**

[1] B. Fu, X. Dong, X. Yu, Z. Zhang, L. Sun, W. Zhu, X. Liang, H. Xu, *New J. Chem.* **2021**, *45*, 2141-2146.

[2] N. R. Cichowicz, P. Nagorny, *Org. Lett.* **2012**, *14*, 1058-1061.

[3] Y. Lin, Y. Zhang, J. Zhang, M. Marcinskas, T. Malinauskas, A. Magomedov, M. I. Nugraha, D. Kaltsas, D. R. Naphade, G. T. Harrison, A. El‐Labban, S. Barlow, S. De Wolf, E. Wang, I. McCulloch, L. Tsetseris, V. Getautis, S. R. Marder, T. D. Anthopoulos, *Adv. Energy Mater.* **2022**, *12*, 2202503.

[4] A. D. Beck, *J. Chem. Phys.* **1993**, *98*, 5648-5646.

[5] F. Weigend, R. Ahlrichs, *Phys. Chem. Chem. Phys.* **2005**, *7*, 3297-3305.

[6] M. Frisch, G. Trucks, H. Schlegel, G. Scuseria, M. Robb, J. Cheeseman, G. Scalmani, V. Barone, G. Petersson, H. Nakatsuji, *Inc., Wallin* **2016**, *248*.

[7] J. P. Perdew, K. Burke, M. Ernzerhof, *Phys. Rev. Lett.* **1996**, *77*, 3865.

[8] a) G. Kresse, D. Joubert, *Phys. Rev. B* **1999**, *59*, 1758; b) P. E. Blöchl, *Phys. Rev. B* **1994**, *50*, 17953.

[9] a) G. Kresse, J. Hafner, *Phys. Rev. B* **1993**, *48*, 13115; b) G. Kresse, J. Furthmüller, *Phys. Rev. B* **1996**, *54*, 11169.
